# A 14^th^ century CE *Brucella melitensis* genome and the recent expansion of the Western Mediterranean clade

**DOI:** 10.1371/journal.ppat.1011538

**Published:** 2023-07-31

**Authors:** George S. Long, Jessica Hider, Ana T. Duggan, Jennifer Klunk, Katherine Eaton, Emil Karpinski, Valentina Giuffra, Luca Ventura, Tracy L. Prowse, Antonio Fornaciari, Gino Fornaciari, Edward C. Holmes, G. Brian Golding, Hendrik N. Poinar

**Affiliations:** 1 Department of Biology, McMaster University, Hamilton, Canada; 2 McMaster Ancient DNA Centre, Departments of Anthropology and Biochemistry, McMaster University, Hamilton, Canada; 3 Department of Anthropology, McMaster University, Hamilton, Canada; 4 Daicel Arbor Biosciences, Ann Arbor, Michigan, United States of America; 5 Division of Paleopathology, Department of Translational Research and New Technologies in Medicine and Surgery, University of Pisa, Pisa, Italy; 6 Department of Biotechnological and Applied Clinical Sciences, University of L’Aquila, L’Aquila, Italy; 7 Division of Pathology, San Salvatore Hospital, Coppito, Italy; 8 Maria Luisa di Borbone Academy, Viareggio, Italy; 9 Sydney Institute for Infectious Diseases, School of Medical Sciences, University of Sydney, Sydney, Australia; 10 Department of Biochemistry, McMaster University, Hamilton, Canada; 11 Michael G. DeGroote Institute for Infectious Disease Research, McMaster University, Hamilton, Canada; 12 CIFAR Humans and the Microbiome Program, Toronto, Canada; University of Mississippi Medical Center, UNITED STATES

## Abstract

Brucellosis is a disease caused by the bacterium *Brucella* and typically transmitted through contact with infected ruminants. It is one of the most common chronic zoonotic diseases and of particular interest to public health agencies. Despite its well-known transmission history and characteristic symptoms, we lack a more complete understanding of the evolutionary history of its best-known species—*Brucella melitensis*. To address this knowledge gap we fortuitously found, sequenced and assembled a high-quality ancient *B. melitensis* draft genome from the kidney stone of a 14^th^-century Italian friar. The ancient strain contained fewer core genes than modern *B. melitensis* isolates, carried a complete complement of virulence genes, and did not contain any indication of significant antimicrobial resistances. The ancient *B. melitensis* genome fell as a basal sister lineage to a subgroup of *B. melitensis* strains within the Western Mediterranean phylogenetic group, with a short branch length indicative of its earlier sampling time, along with a similar gene content. By calibrating the molecular clock we suggest that the speciation event between *B. melitensis* and *B. abortus* is contemporaneous with the estimated time frame for the domestication of both sheep and goats. These results confirm the existence of the Western Mediterranean clade as a separate group in the 14^th^ CE and suggest that its divergence was due to human and ruminant co-migration.

## Author summary

We describe the reconstruction of a high-quality ancient *B. melitensis* genome isolated from the kidney stone of a 14^th^ C. CE Italian friar. We confirm the presence of a *B. melitensis* genome from the Western Mediterranean clade in Italy during this time period and refine previous estimates for the diversification of this phylogenetic group. In addition, we propose that the speciation event separating *B. abortus* and *B. melitensis* occurred during the domestication of sheep and goats approximately 9000 BCE.

By comparing the gene content of the ancient *B. melitensis* genome with modern isolates, we provide evidence that neither the *B. melitensis* phylogenetic groups nor their respective sequence types necessarily correspond with the genetic components of their strains. The accessory genome of *B. melitensis* appears to be influenced by its geographic location, more so than its sequence type. We propose splitting the Eastern Mediterranean clade into three smaller groups to better reflect these genomic differences.

## Introduction

Brucellosis is a zoonotic disease caused by the bacterium *Brucella* that is most often transmitted to humans by domestic livestock through the consumption of animal products and the interactions with infected animals [1]. It is one of the most common zoonotic diseases with an estimated 500,000 cases each year, although its incidence is hypothesized to be far higher (∼ 5 to 12.5 million) [2, 3]. Three main species of *Brucella* are pathogenic to humans. *B. melitensis* is carried by sheep and goats while *B. suis* and *B. abortus* are found in pigs and cows, respectively [4]. Brucellosis is a serious infection causing fever, chills, extreme muscle pain, and long-term osteoarticular changes that can be observed on bones [1]. As a chronic disease it is of particular importance to public health due to its indirect, yet significant economic impacts, by decreasing the workforce and reducing livestock reproduction and their associated products [5]. Brucellosis is common today in Asia, the Middle East, Africa, South America, and the Mediterranean [3]. Southern European countries make up the bulk of brucellosis infections in Europe, with Greece accounting for 20.3% of human cases while Italy has the highest prevalence of cattle herds, sheep and goat flocks testing positive for *Brucella* within the European Union as of 2020 [6].

Brucellosis is thought to have been a common scourge in the Mediterranean in the past, especially during the Roman and Medieval periods before the advent of pasteurization, and when pastoral practices such as transhumance were commonplace [7, 8]. However, little historical or archaeological evidence exists to support this claim [9]. Purported cases from the Roman period were identified in 16 skeletons from Herculaneum dating to 79 CE through the presence of non-specific vertebral lesions [8]. While skeletal lesions are a common manifestation of brucellosis, occurring in 10 to 85% of modern clinical cases, the lesions tend to occur in similar locations and with a similar appearance as those caused by tuberculosis [10], leading to potential confusion in differentiating between the two diseases in the past. If the pattern of lesion occurrence in brucellosis cases was the same as it is today, we would expect that more differential diagnoses of brucellosis would be reported from ancient remains [10]. However, there are several reasons why brucellosis may be under-reported in paleopathological analyses of archaeological samples; it is likely mistaken for other diseases that similarly impact bone like tuberculosis, the bacterium had not left skeletal changes by the time of death [7, 11, 12], or the non-specific nature of the lesions precluded a diagnosis [12].

Support for the presence of brucellosis during the Medieval period includes an ancient genome identified from a pelvic nodule of an individual in Sardinia dating to *c*. 1300 CE; two individuals from Albania (*c*. 900—*c*. 1200 CE) with PCR products arising from lesioned ribs and vertebrae; and two French individuals from the 14^th^ and 18^th^ C. CE where an infection was identified via paleopathology [13–15]. This limited DNA evidence compared to other infectious diseases could be due to improper sample selection [16], poor DNA preservation [12, 17, 18], or its mistaken diagnosis [18]. It is also possible that a focus on other diseases such as tuberculosis has meant that researchers have neglected possible evidence for brucellosis in the archaeological record.

The Mediterranean region is thought to have been a common geographic location for the origin and divergence of all *Brucella* species (especially *B. melitensis*) due to the diversity of strains in this area and the basal position of the Western Mediterranean clade in the global *Brucella* phylogeny [19, 20]. Despite this, the evolutionary history of the disease in the region is poorly understood. Due to the widespread movement of infected animals across regional, national, and international borders, the phylogeography of *Brucella* on both local and larger scales has been challenging to elucidate using modern isolates [19]. Sheep and goats are the preferred hosts for *B. melitensis*, and while spillover infections can occur, these alternative hosts tend to result in dead-end infections [4, 21]. This is especially the case in some animals that require constant exposure to *B. melitensis* to sustain an infection [22]. Sheep and goat husbandry extended across the Mediterranean littoral zone (Cyprus to Atlantic Portugal) by 5000 BCE [23–25]. Based on the dates of expansion of ruminants into the region during the Neolithic and dates of divergence for *B. melitensis*, it is hypothesized that the bacterium entered the region with the introduction of ruminants and subsequently diversified [4, 26, 27]. Paleopathological diagnoses of brucellosis in human skeletal remains from Jordan and Palestine (2100–1550 BCE), and Bahrain (3000–1200 BCE) support this hypothesis [7, 28, 29].

Archaeological and genetic data on the spread of sheep into the Mediterranean illustrate the complexity of repeated expansions and contractions over multiple periods of domestication. Subsequent selective breeding, trade, and geographic expansion have impacted the genetic signatures of sheep causing them to have little resemblance to their early progenitors [25, 30, 31]. Wild sheep and goats were domesticated approximately 10,000–8000 BCE in the Fertile Crescent [25, 32–34]. First domesticated for meat, sheep were later kept for their secondary products such as milk and wool [33]. The first expansion of domesticated sheep and goats from the Zagros mountains (northwestern Iran to southeastern Türkiye) spread to the borders of Europe and Africa by 8000 BCE [25, 33] following two routes of expansion; the Danubian route ran from Türkiye through the Balkans to the interior of Europe, while a southern route went through North Africa (North; Türkiye, Calabria, Corsica, and France; South—Iraq, Jordan, Egypt, Morocco) [25, 34, 35].

Subsequent expansions related to secondary features (*e.g*., fat tailed sheep, wool sheep) occurred between 4000 to 3000 BCE in Europe [32, 33, 35]. Wool sheep likely originated in Southwest Asia, spread west and then across Europe from the Iberian Peninsula [30, 35]. Wool producing sheep partially replaced the initial domesticated sheep (hair sheep) and earlier breeds derived from previous introductions via different routes (Mediterranean versus Danubian). Evidence for this replacement is supported by a larger number of ancestral SNPs in modern domesticated sheep from southeastern to northwestern Europe (i.e., the proportion of ancestral genetic components decrease when one is further away from the Fertile Crescent) [30, 32]. Italy and the Balkans were hubs for migrations of early and late domesticates such as wool sheep [32, 35].

An acute lack of genome-scale data concerning *B. melitensis* has impeded our understanding of its origin, evolution, and spread. Until recently, public databases contained few *B. melitensis* genomes, limiting inferences on epidemiological history. This lack of data has since changed due to the recent focus on the Western Mediterranean clade of the bacterium in several studies [20, 27, 36]. Although it was hypothesized that this clade has a long-standing presence in the Mediterranean it was underrepresented in genomic databases [26]. A recent study increased the representation of the diversity within Western Mediterranean clade by adding 339 Italian strains from humans and ruminants to public databases [26]. A lack of public health support for genome surveillance may also explain in part why other regions, such as Africa, are similarly under-sampled [37, 38].

In contrast to the paucity of genome-scale data, the distribution of sequence types (ST)—the allelic profile of core housekeeping genes in a bacterial species—within *B. melitensis* is well defined [39, 40]. Sequence types provide a more nuanced view of the diversity within the *Brucella* genus. These STs can be used to quickly identify the phylogenetic group of an unknown *B. melitensis* strain as they are relatively well conserved. For example, there are five STs (ST 9, 11, 87, 88, and 89) present in the Western Mediterranean clade [40]. Biovars—groups of isolates divided by their biochemical properties—were previously used to categorize *Brucella* strains. The Western and Eastern Mediterranean phylogenetic groups, however, contain genomes arising from all three known *B. melitensis* biovars, indicating that STs are superior for discriminating between strains [40].

Only one partial ancient *B. melitensis* genome has been reconstructed to date, although its coverage is unfortunately too low to allow for genome scale analyses and comparison here (see S1 Appendix) [13]. The lack of ancient genomes impedes analyses attempting to determine the most recent common ancestor of extant *B. melitensis* as well as its overall phylogeographic history and gene content. Here, we describe the successful reconstruction of a high quality *B. melitensis* genome from the kidney stone of a beatified 14^th^ CE friar, Sante Brancorsini (1343—1394 CE). The high resolution and precise date of this new genome along with its geographic location and associated time period make it particularly useful for addressing questions about the evolution of *B. melitensis* in the Mediterranean.

## Results

### Paleopathological overview

A paleopathological survey of the remains of Sante Brancorsini revealed several vertebrae with lesions characteristic of brucellosis [41]. The cavities caused by these lesions may have also caused spondylodiscitis—the inflammation of the nucleus pulposus—which while not typical of brucellosis, has also been reported [42]. Several calcified nodules were identified in the abdomen, which were later identified to be calcified kidney stones [43]. A more detailed look at the paleopathological examinations of Sante Brancorsini can be found in the original study [16].

### Validating the samples

A metagenomic analysis of DNA reads from the nodule (S1 Fig) libraries showed that a substantial fraction of the classified reads stemmed from the genus *Brucella* (2,777,352 reads or 19.501%) and *Homo sapiens* (8,576,720 or 67.740%). *Bradyrhizobium* was the dominant taxa identified in extraction blanks (1869 reads or ∼ 25.363%), with *Brucella* being present in exceptionally low amounts (2 reads or ∼ 0.03%) (S2 Fig).

**Fig 1:**
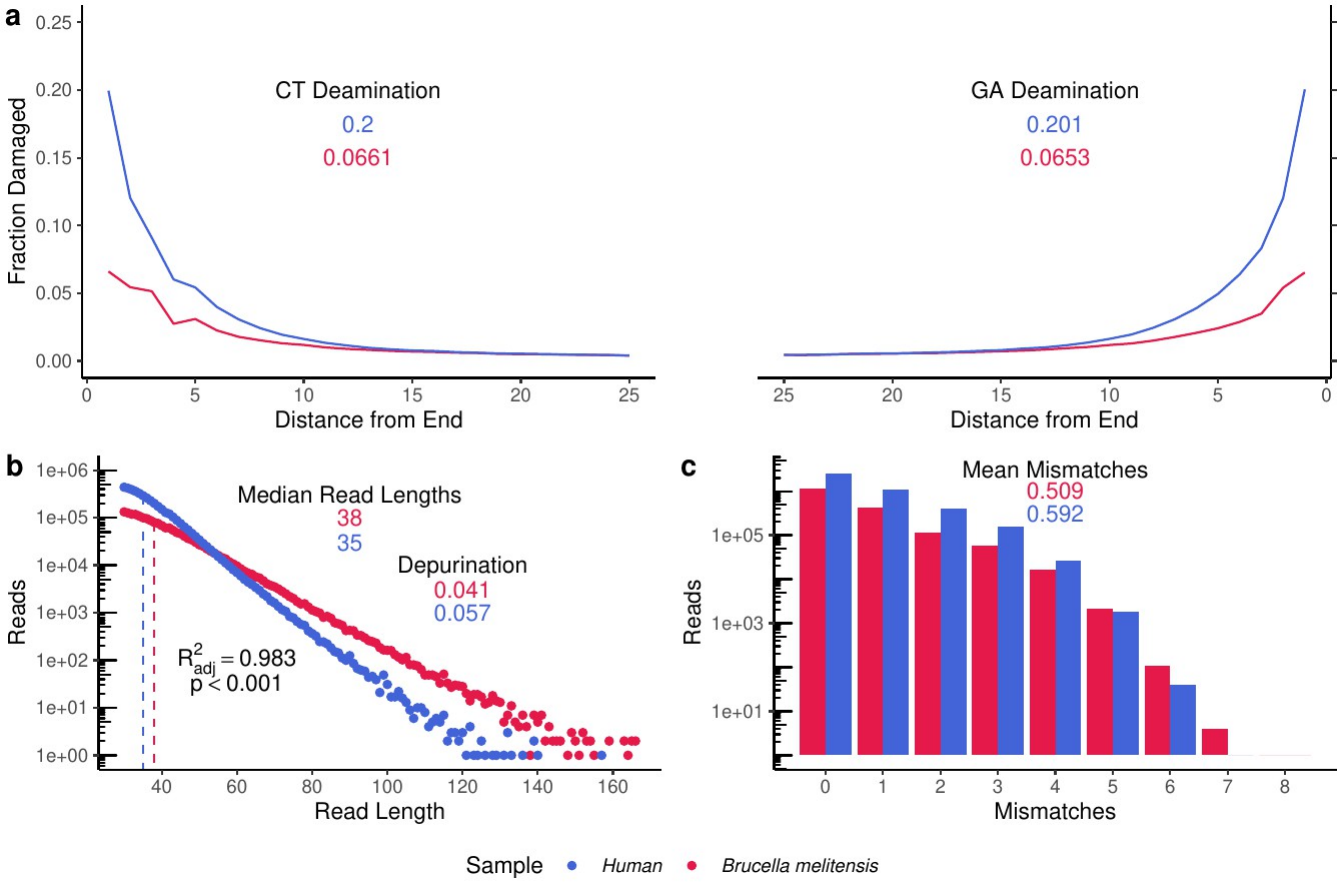
DNA authentication of the ancient *B. melitensis* genome. **a)** Mapdamage plot showing the fraction of reads with deamination present on the 5′ (left) and 3′ (right) ends of the molecules. A hard masked hg38 human and the reference *B. melitensis* genomes were used to create this plot. **b)** FLDs of the reads mapped against their respective genomes. **c)** Edit distance plot for the mapped reads.

The deamination pattern for reads mapping to *B. melitensis* appear to indicate authentic and relatively well preserved ancient DNA compared to the human DNA isolated from the same nodule (Fig 1a). This difference in preservation is supported by the fragment length distribution of the DNA reads, where *B. melitensis* has a longer median read length and a significantly (linear regression: *p* < 0.001) shallower slope than its associated human DNA (Fig 1b). The edit distance distributions also support its authenticity, with the greater mean mismatch count for the human genome potentially caused by the higher reported deamination and other damage signals (Fig 1c). Discrepancies in preservation rates between the host and pathogen of interest are not a new phenomenon [44, 45] and it has been reported that GC content is a likely driver of depurination rates [46]. The difference in preservation rates were likely also magnified by the presence of a cell wall in *B. melitensis* [47].

### *B. melitensis* genome reconstruction and phylogenetic analysis

**Fig 2:**
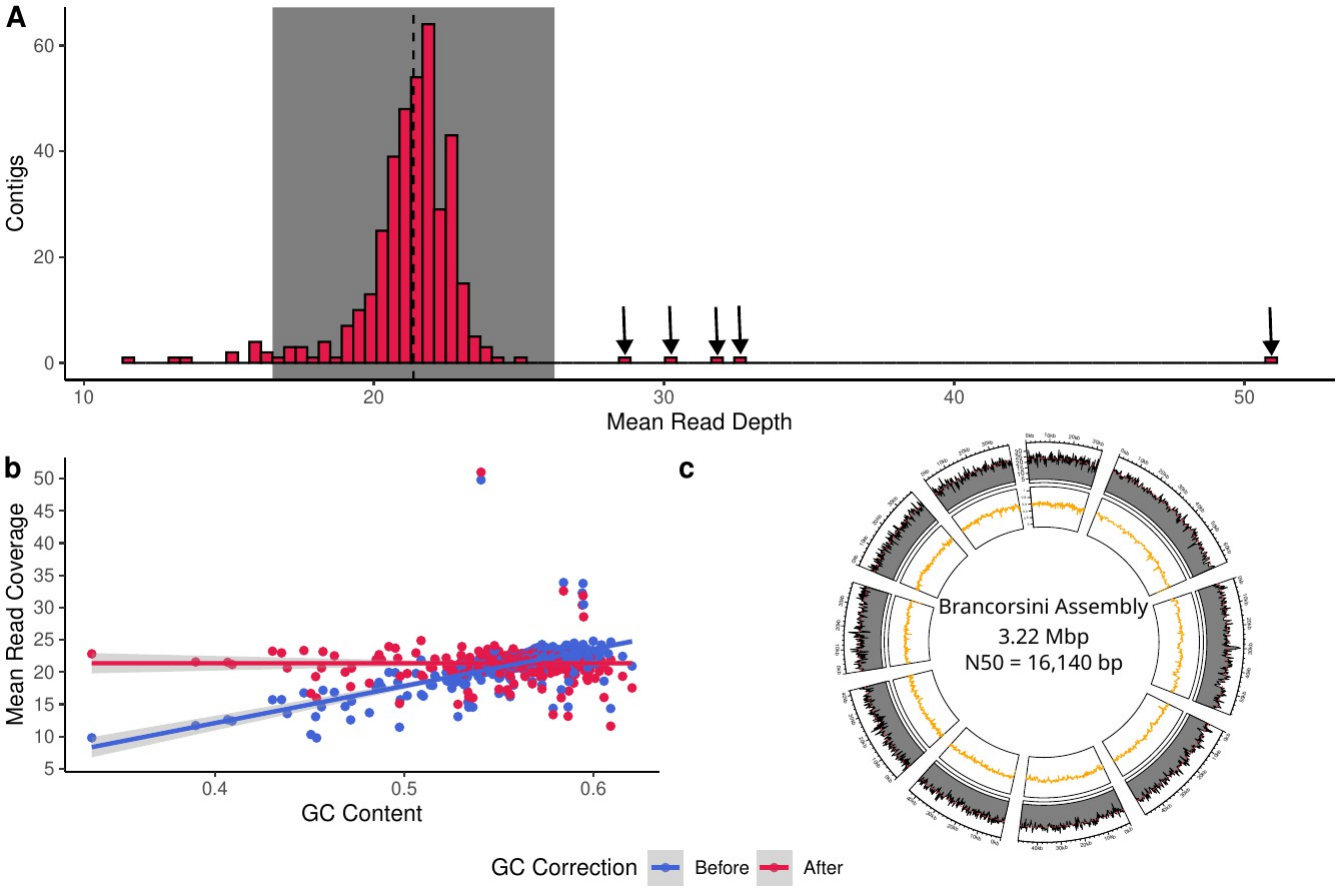
Read coverage metrics of the *B. melitensis* pan-genome. **a)** Distribution of mean contig coverages of the ancient *B. melitensis* assembly. The dashed line indicates the mean coverage while the shaded rectangle indicates ±2 standard deviations from the mean. Arrows indicate contigs with a significantly greater read depth. **b)** GC bias correction results for the mapped contigs. **c)** Circos plot of the ten largest contigs in the ancient *B. melitensis* assembly. The outer ring indicates the mean read depth over a 0.5% sliding window whereas the inner ring is the GC content over the same period.

A total of 2996 genes were identified in the ancient *B. melitensis* assembly which had an overall mean coverage of 21.37 [21.13, 21.61]× (Fig 2a, Table 1). While GC correction did improve results (Fig 2b), five of the contigs still had significantly (two-sided t-test: *p* < 0.001) deeper read depths than the rest of the assembly. A search for the genes associated with these higher copy number contigs identified the elongation factor *tuf* (*tuf_1* in the pan-genome), an IS5 family transposase (*group_1* in the pan-genome), and a porin related protein (*group_1246* in the pan-genome) indicating that first two genes could potentially be transposons responsible for relocating the latter. The other two higher copy number contigs corresponded to fragments of an IS5 element and a tRNA gene per a blastX search of the RefSeq protein database (S1 File).

**Table 1:** Summary Statistics of the ancient *B. melitensis* assembly. An overview of the ancient *B. melitensis* assembly. The square brackets represent the 95% confidence interval.

Metric	*denovo* Assembly
**Contigs**	386
**Assembly Length** (bp)	3, 220, 175
**N50** (bp)	16, 140
**Mean Contig Depth** (×)	21.37 [21.13, 21.61]
**GC Content** (%)	56.1 [55.8, 56.5]
**Genes Identified**	2996
**Core Genes Identified**	2826
**Proportion of Core** (%)	97.5

It is unlikely that these contigs originate from a phage or a plasmid as *Brucella* has been repeatedly shown to have a closed genome [48–50], furthermore, a search of the contigs with MOB-suite [51] did not identify any mobile elements. *Brucella* has not exhibit any signs of having recently undergone heterologous recombination [49, 50], thus the five higher copy number contigs must represent duplicated regions in the chromosome. This can be confirmed through a blast search of the nt database as there were multiple matches of these five contigs to the same accession (S1 File). Despite these outliers the overall coverage of the contigs is even with very few regions lacking reads (Fig 2c).

**Fig 3:**
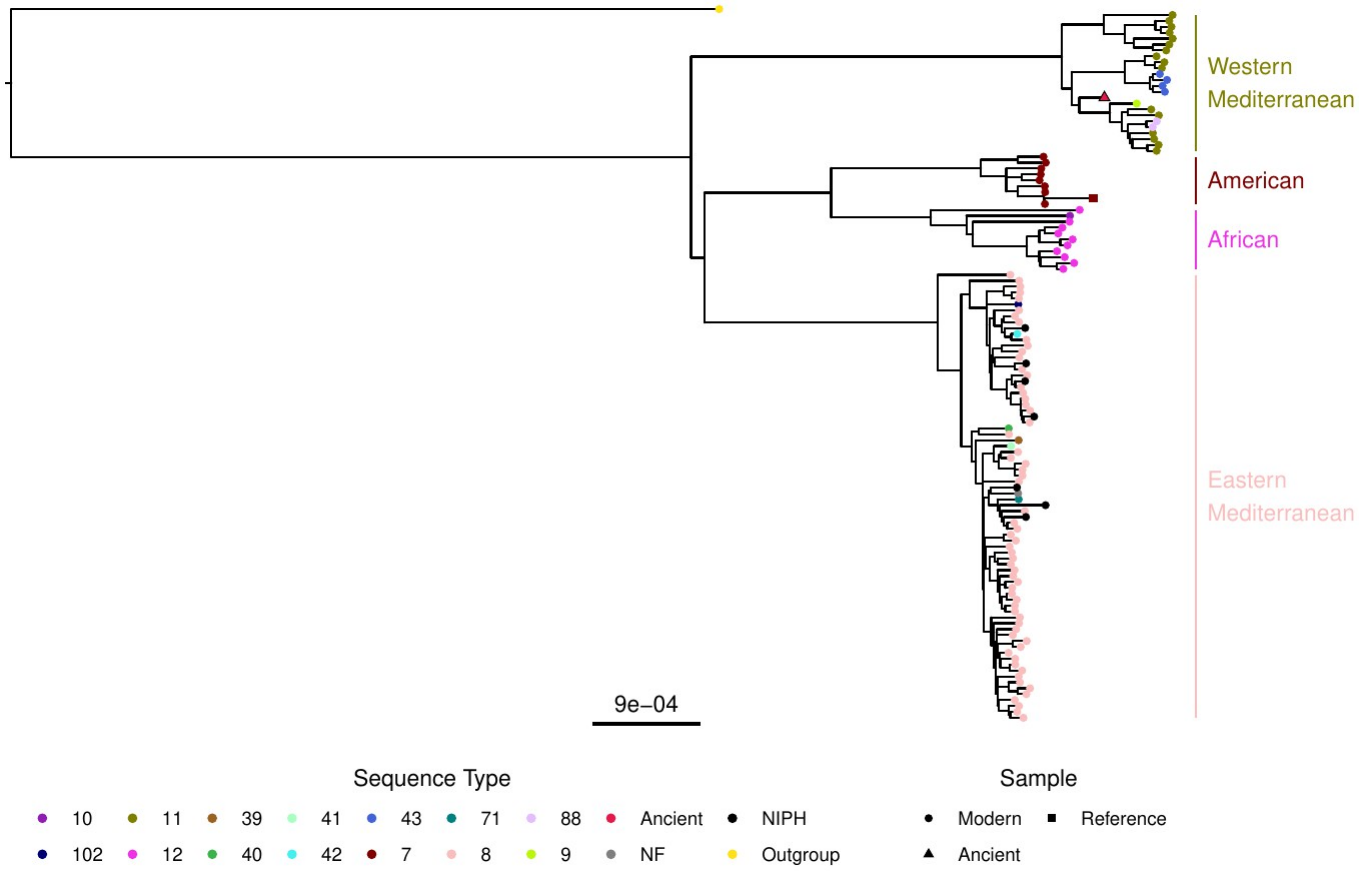
Maximum Likelihood Phylogeny of *B. melitensis*. Core SNP maximum likelihood phylogeny of the global distribution of *B. melitensis*. *B. abortus* 2308 was the outgroup used to root the tree while *B. melitensis* 16M is the reference genome.

Maximum likelihood (ML) phylogenies were estimated for both the global and Western Mediterranean *B. melitensis* genome populations using 323 genomes obtained from NCBI on 2019–09-17. The global phylogeny—which used a trimmed dataset containing 17, 510 core SNPs—firmly placed the ancient genome within the Western Mediterranean clade (Fig 3) recapitulating previously published trees [20, 26]. While the ancient strain is not ancestral to the entire phylogenetic group it is positioned basal to a specific sub-clade, indicating that these sub-groups were present in the 14^th^ century. No temporal signal was present (linear regression: $p=0.740,R_{adj}^{2}=-0.007$) in the global phylogeny when using TempEst [52].

**Fig 4:**
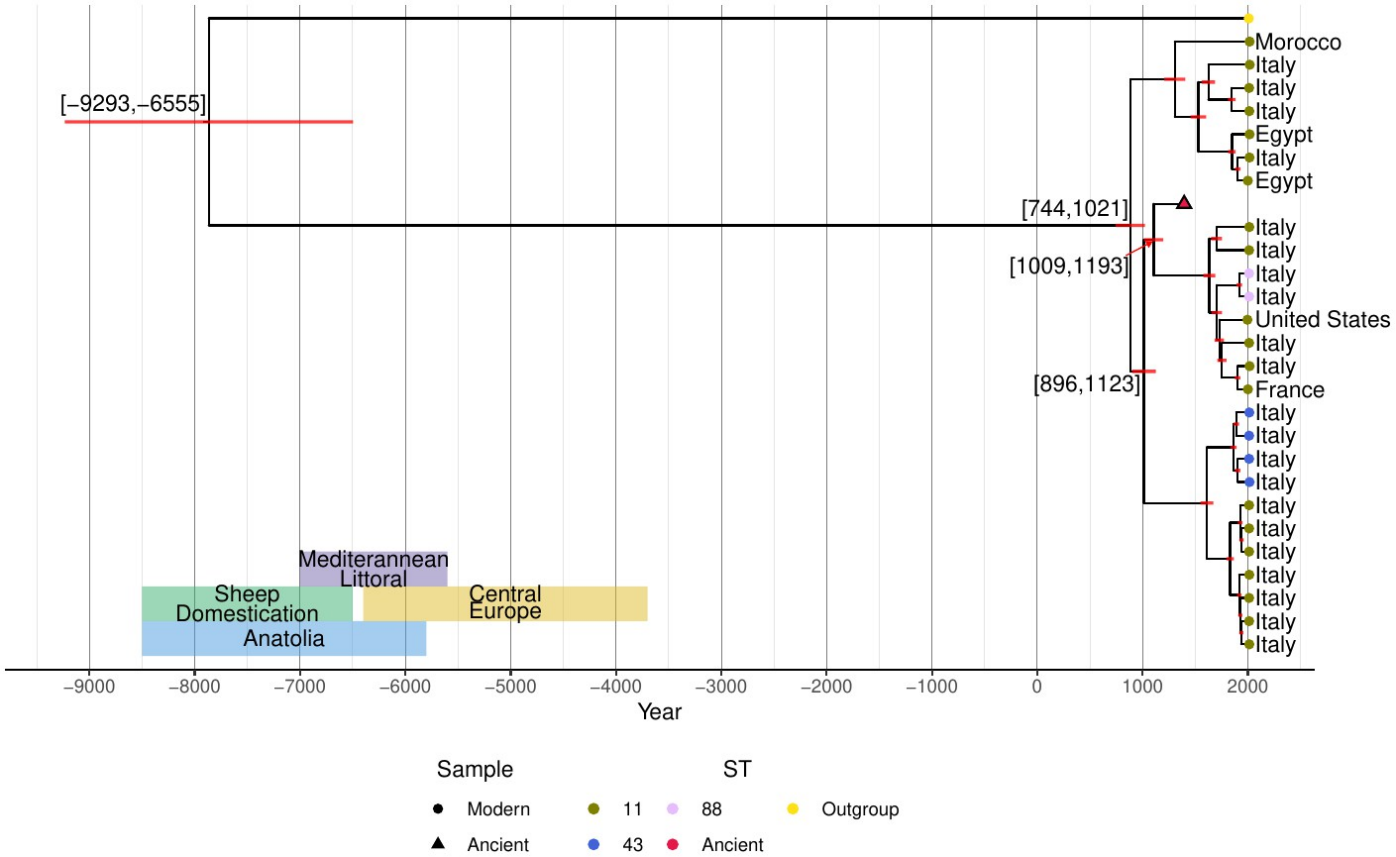
Phylogenetic analysis of the Western Mediterranean clade. Core SNP Bayesian phylogeny of the Western Mediterranean phylogenetic group. The red bars at the nodes indicate the 95% highest posterior density of the estimated date for a node whereas the text indicates the boundaries of the interval. The text along with coloured shaded regions indicate the estimated dates for sheep domestication and migration to specific regions [105]. When available, the genomes are labelled by the their country of isolation. *B. abortus* 2308 was the outgroup and reference genome.

We next performed a similar analysis of only the Western Mediterranean clade phylogeny using an alignment of 8262 core SNPs (Fig 4 and S5 Fig). The ancient genome fell in the same position as it did within the global phylogeny, and in this case there was strong evidence for a temporal (i.e. molecular clock) signal in both the ML (linear regression: $p<0.001,R_{adj}^{2}=0.76$) and Bayesian phylogenies (log Bayes factor: 59.75). Under a strict molecular clock, the mean nucleotide substitution rate for the Western Mediterranean clade was 9.322 ⋅ 10^−8^ [8.062 ⋅ 10^−8^, 1.062 ⋅ 10^−7^] subs/site/year, giving a mean divergence date of 1105 [1009, 1193] CE for the ancient strain. Under this model the diversification of the Western Mediterranean clade occurred about 884 [744, 1021] CE and that speciation from *B. abortus* likely happened about 7865 [9293, 6555] BCE. We obtained very similar results using a relaxed molecular clock (i.e. a nucleotide substitution rate of 9.463 ⋅ 10^−8^ [7.625 ⋅ 10^−8^, 1.134 ⋅ 10^−7^] subs/site/year) suggesting that these estimates are robust to rate variation.

### Functional analysis

The majority of the genes identified in the ancient strain (2826 of 2966) stemmed from the core genome of *B. melitensis*. Despite this, 72 core genes were absent from the ancient strain. A COG database search identified the functional categories for most of the missing core genes with a large number involved in metabolism or biogenesis (Table 2). These missing genes may have impacted the virulence of the ancient strain. Several of these genes were involved in the *Brucella* virulence related regulatory and sensory (BvrR/BvrS) mechanism responsible for directing the expression of several outer membrane proteins [53, 54]. The same results were also found with a STRING [55] database search (S1 File). A similar search for known *B. melitensis* virulence factors [56] was also performed, revealing that the ancient genome was likely pathogenic as genes from each virulence category were detected in the ancient strain (Table 3). AMR genes were also analyzed using the CARD database [57], although only four were identified in the ancient genome (Table 4) three of which were efflux pumps. Thus, the ancient genome did not contain any significant resistance mechanisms [58, 59].

**Table 2:** Identified COG categories of core genes absent from our ancient strain. Genes were identified through a blastX search against the 2020 COG database. The gene counts are not exclusive as a gene may be identified in multiple categories.

Function	Gene Count
Cell wall/membrane/envelope biogenesis	8
Lipid transport and metabolism	5
Translation, ribosomal structure and biogenesis	4
Amino acid transport and metabolism	3
Replication, recombination and repair	3
Function unknown	3
Signal transduction mechanisms	3
Nucleotide transport and metabolism	2
Carbohydrate transport and metabolism	2
Coenzyme transport and metabolism	2
Transcription	2
General function prediction only	2
Posttranslational modification, protein turnover, chaperones	1
Secondary metabolites biosynthesis, transport and catabolism	1
Defense mechanisms	1
Mobilome: prophages, transposons	1

**Table 3:** *Brucella* virulence factors present in our ancient strain. Genes were found through a combination of gene name searches, blasting the COG database, and blasting against a specific gene.

Virulence Category	Genes
Urease	40
Cytochrome Oxidase	11
Lipopolysaccharide	11
Type IV Secretion System	10
BvrR/BvrS System	9
Superoxide Dismutase and Catalase	3
Alkyl Hydroperoxide Reductase	2
Cyclic *β*-1–2-glucans	2
Nitric Oxide Reductase	2
Base Excision Repair	2
Brucella Virulence Factor A	1

**Table 4:** *Brucella* antibiotic resistance genes. Genes were identified by searching the protein homolog models from the CARD database [57]. Gene name indicates the label assigned by Roary [60] whereas AMR Gene is the corresponding gene in the CARD database.

Gene Name	AMR Gene	ARO ID	Resistance Mechanism
group_2536	*mdtP*	3003550	Antibiotic efflux
group_88	*tetA*	3004639	Antibiotic efflux
group_970	*KpnH*	3004597	Antibiotic efflux
*mprF*	*mprF*	3003772	Antibiotic target alteration

**Fig 5:**
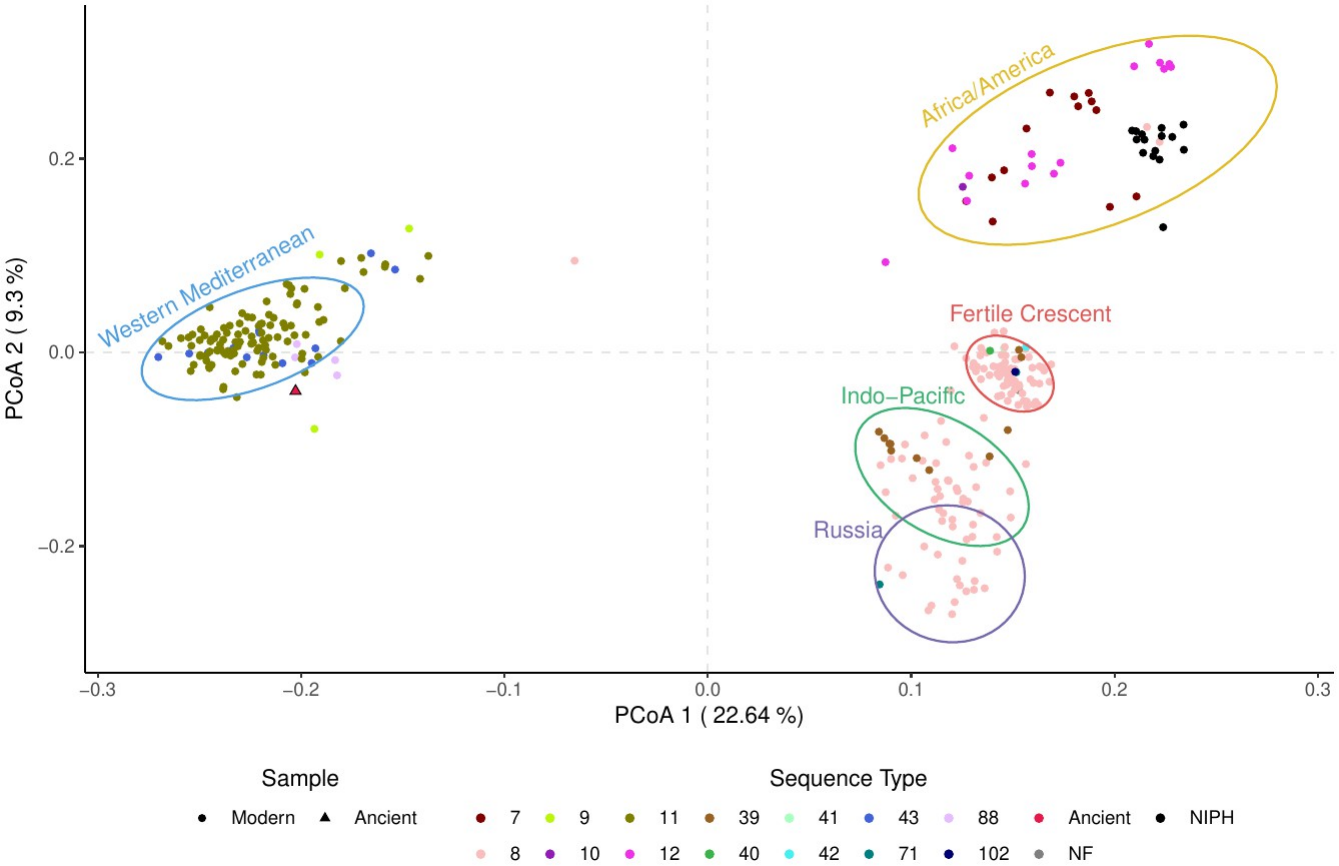
Principal Coordinate Analyses (PCoA) of accessory gene PAV. Sequence types are identified using SRST2, although the outlier ST 8 genomes (from NIPH) are in black. The genomes were also naively clustered using the clara algorithm with the number of clusters selected by searching for the local maximum in the silhouette plots.

The presence-absence variation (PAV) analysis of the accessory genome across modern and ancient genomes recapitulates the results of our phylogenetic analyses (Fig 5). Modern *B. melitensis* accessory genomes grouped into their respective geographic regions (Africa, the Americas, Eastern and Western Mediterranean). The ancient strain was similarly placed close to the Western Mediterranean phylogenetic group, again suggesting that it was an early member of this group. However, an exception to this discrete grouping was the intermixing of the American and African phylogenetic groups. This mixture of groups also supported by a SNP multiple correspondence analysis (MCA) suggests that these two phylogenetic groups are not representative of distinct functional differences (S7 Fig). The same reasoning applies to the wayward sequence type 8 (ST) genomes clustering with the African and American strains. These strains were first identified as part of a Norwegian Institute of Public Health (NIPH) study analyzing the breadth of *B. melitensis* cases reported in the country [61]. They form a group of infections originating from Afghanistan, Georgia, Iraq, Norway, Syria, and Türkiye. While both their ST and core SNP phylogeny indicate that these strains are members of the Eastern Mediterranean clade, the PAV of the accessory genome places them within the African and American cluster. This further suggests that STs and phylogenetic clades do not convey sufficient information to convey functional differences between strains.

**Fig 6:**
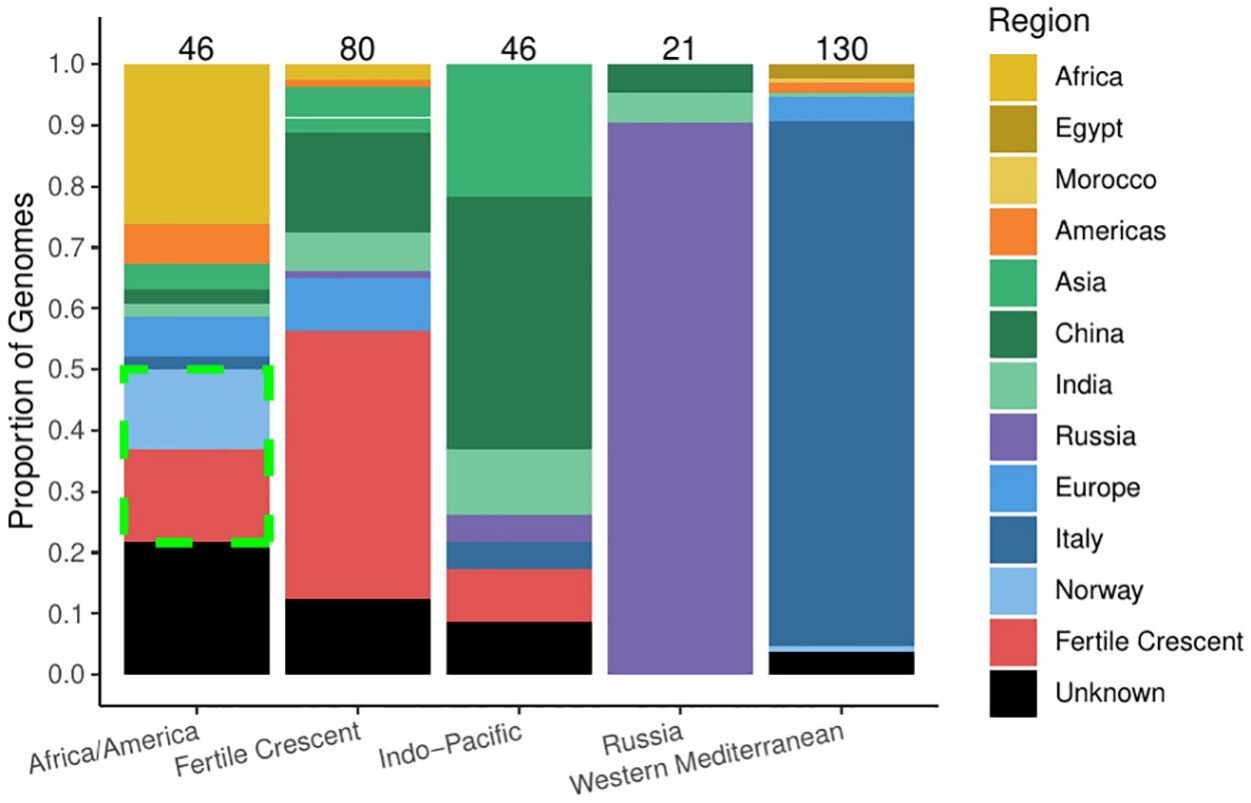
Isolation sources for the naive accessory gene PAV clusters. Colours for the regions correspond with the PCoA clusters. The wayward ST 8 genomes are indicated by dashed green lines.

Naively clustering *B. melitensis* accessory genome strains (PAV) based on a principal coordinate analysis (PCoA) showed that the Eastern Mediterranean clade formed three distinct clusters (Fig 6). The first of these clusters contains the majority of genomes originating from the Fertile Crescent and is also primarily part of a sub-clade of the Eastern Mediterranean phylogenetic group in the core SNP phylogeny. There is substantial spillover of this cluster with the rest of the phylogeny, although they largely consist of genomes isolated outside of the Fertile Crescent (*i.e*. China and India). As it is hypothesized that *B. melitensis* originated within the Fertile Crescent [26], these results are of note as they suggest the presence of a distinct, stable population at its starting point. Also, the wayward ST 8 genomes in the African/American cluster are phylogenetically placed with the Fertile Crescent strains in the global core SNP phylogeny and form a sub-cluster within the African/American group. That most of these wayward strains were associated with infections in the Fertile Crescent suggests that loss of ten specific accessory genes is enough to be functionally similar to the African/American group (S1 File, S8 Fig). The other two clusters represent the remaining Eastern Mediterranean phylogenetic group, with one representing the Indo-Pacific region while the other consisting primarily of genomes isolated from Russia.

The five genome clusters are the result of the PAV of a relatively small number of accessory genes (Table 5, S8 Fig). These genes are uniquely present or absent in their respective geographic clusters, and thus have an overly large impact on the positioning of the genomes in the PCoA. This is true regardless of whether or not the genes are filtered by their overall presence in the species. Furthermore, these genes are enough to impart additional gains or loss of function to the genomes (S1 File). The Russian and Western Mediterranean clusters are prime examples as they contain a large number of genes uniquely present in their genomes. The genes present in the Western Mediterranean impart a unique set of transferases and while the Russian strains have a separate set of 30S ribosomal proteins and metal transporters (S1 File). The presence of these genes may be sufficient to determine the geographic region of a strain, especially given the clonal nature of *Brucella* [39].

**Table 5:** Number of accessory genes uniquely present or absent in the accessory genome clusters. Results are shown for both the entire set of accessory genes as well as those present in at least five genomes.

Region	All Accessory Genes		Genes in ≥5 Genomes	
	Genes Absent	Genes Present	Genes Absent	Genes Present
Africa/America	6	20	6	10
Fertile Crescent	5	4	5	1
Indo-Pacific	0	20	0	0
Russia	8	109	8	36
Western Mediterranean	10	44	10	22

## Discussion

### Authenticating the ancient *B. melitensis* genome

Metagenomic analyses indicated that the kidney stone from Sante Brancorsini contained *Brucella sp*. DNA (S2 Fig). The DNA was confirmed to arise from *B. melitensis* due to the high and relatively even coverage of reads across the assembly (21.37 [21.13, 21.61]x) (Fig 2a and 2c) and low number of mismatches (*i.e*, edit distance) when mapping against a *B. melitensis* (GCF_000007125) genome (Fig 1c and S9 Fig). Furthermore, approximately 92% of the recovered genes stem from the *Brucella* core genome.

In addition, the ancient *B. melitensis* and human DNA reads possess clear signals of deamination (Fig 1a) and depurination (Fig 1b) characteristic of ancient DNA damage. The fragment lengths in the mapped reads form an exponential distribution as expected (Fig 1b) [62].

*Brucella* is not a commensal genus of bacteria [63] and our identification of key virulence genes [50, 64, 65] confirms that the ancient genome was indeed pathogenic. The strain lacked resistance genes apart from the detection of efflux pumps (Table 4). Multi-drug efflux pumps, while also being involved in other mechanisms, do not provide strong antibiotic resistance on their own [50, 58, 59]. Modern *Brucella* genomes, while rare, can demonstrate signs of resistance to azithromycin [66], rifampicin [61], streptomycin, sylfamethoxazole, and ceftriaxone while susceptibility is decreasing for other antimicrobials [67].

### Time-scaled speciation of *Brucella*

The presence of several *B. melitensis* sequence types circulating in the modern Mediterranean littoral [19, 20, 26, 27] has led to the hypothesis that *B. melitensis* either originated in this region or was endemic, especially in Italy, for an extended period of time [19, 20]. The results of our Western Mediterranean *B. melitensis* phylogeny support the latter theory, which has also been supported by other recent studies [26, 27]. Our speciation date of *B. melitensis* is consistent with previously published dates of sheep and goat domestication in the Fertile Crescent [25]. Furthermore, with previous studies indicating that the Western Mediterranean clade diverged during the 4^th^ or 5^th^ millennium BCE [26, 33] it is likely that *B. melitensis* arose in the Fertile Crescent and was brought to the Western Mediterranean during the shift from hunter-gather practices to pastoralism [31, 33, 35, 68].

Although the process of moving from a hunter-gatherer society to an agriculturalist culture was complex, the spread was most often a shift to agro-pastoralism in the coastal regions of the Mediterranean with several colonies sprouting across the region. In contrast, the spread of the Neolithic package [25, 69, 70] was much slower in inland locations. Generally, this variation in the speed of adoption meant that the coastal areas were more reliant on domestic livestock whereas the inland regions used a mix of domestication and hunter-gather practices during a similar time [25]. The process of adopting agricultural practices in Europe was completed by the 4^th^ millennium BCE [71] with farming practices intensifying from nomadic pastoralism to transhumance. This transition from mixed hunter-gatherer and agricultural practices including transhumance pastoralism could have impacted the divergence of the Western Mediterranean *B. melitensis* clade during this time.

Specialization in wool sheep occurred between 5000 and 4000 BCE in the Fertile Crescent with the eventual replacement of the first domesticates (hair sheep) beginning approximately 4000 BCE. These sheep eventually spread to Europe sometime between 3000 and 2000 BCE [30, 32, 33, 35, 72]. Wool was a major innovation in Europe during the 2^nd^ millennium BCE. Historical sources state that wool production thrived on the coasts of the northeastern Mediterranean [73]. The spread of wool sheep from Asia to Europe along with the level of production in the latter indicates there was a substantial amount of animal movement in these regions during this time [73]. The movement of wool sheep could have also contributed to the divergence of the Western Mediterranean clade as wool specialization also occurred during the same time as our estimated dates of divergence.

The placement of the *B. melitensis* genome in the Western Mediterranean clade supports the hypothesis of an early expansion starting in the 4^th^ millennium BCE. Such results propose that the migration of sheep and goat populations are the driving force in the diversification of *B. melitensis*. This is especially true given the transportation of sheep and goats across Eurasia throughout their history as domesticates [30, 32, 73]. In combination with the clonal nature of *B. melitensis* [38], its highly conservative genome [48, 50], and the sequence typing results of the ancient genome (S1 Appendix) it is likely the phylogenetic structure is a direct result of these early migrations.

### Potential divisions in the Eastern Mediterranean phylogeny

The PAV of the accessory genes largely recapitulates the global *B. melitensis* phylogeny (Fig 4). The ancient genome shares the same general functions as strains from the Western Mediterranean clade. As *B. melitensis* rarely undergoes horizontal gene transfer [48], these results confirm that the Western Mediterranean clade has remained functionally stable for nearly 700 years (S8 and S10 Figs). The complete complement of virulence genes serves as an example of this long-term stability.

The information gained by analyzing the global phylogeny and the accessory PCoA together provide some insight into the evolution of this genus. A complete separation of the Western Mediterranean strains from all other strains in both analyses corroborates the hypothesis that these genomes were some of the first to diverge. Furthermore, the intermixing of the African and American strains suggests that they originated from a single clade that recently diverged. The Eastern Mediterranean phylogenetic group fell into three clusters that roughly correspond to different geographic regions within this area (Fig 6). The largest of the three clusters is a group of strains primarily from the Fertile Crescent (Iran, Iraq, Israel, Jordan, Kuwait, Syria, and Türkiye) and is of particular interest as it is thought that the practice of sheep husbandry originated in this region [25]. If this is the case, it is possible that this cluster represents the original population and accessory genome content of *B. melitensis*, as these strains have the fewest number of unique and overall accessory genes (S11 Fig). The next largest “Eastern Mediterranean” cluster comprises strains either originating from the Indo-Pacific region with strains primarily from China, India, and Southeast Asia while the final group are genomes from Russia. While the silhouette algorithm fits five clusters, it is likely that the latter two groups (Indo-Pacific and Russia) are part of a larger cluster with global sampling bias artificially splitting them (Fig 6). If not replaced entirely, the “Eastern Mediterranean” group should at least be renamed as to better represent the full extent of its geographic spread.

Of particular interest are the regions of disconnection between the global phylogeny and the accessory genomes. While both data sets agree that the Western Mediterranean clade was the first to diverge from the rest of *B. melitensis* and that the African and American clades are closely related, the Eastern Mediterranean strains complicate this simple scenario. This issue is most obvious with the strains isolated and studied by the NIPH [61]. While these strains cluster with the American and African strains in the PCoA, in the phylogeny they are placed among those from the Fertile Crescent. This disconnect suggests that assigning function to either the ST or the phylogenetic group of strains can be inaccurate and potentially misleading.

The widespread movement of infected animals across regional, national, and international borders make the phylogenetics of *Brucella* challenging at both local and larger scales [19]. Our analyses, using the first high quality ancient *B. melitensis* genome, and applying core SNP and ST methods to compare with the now numerous contemporary genomes, provides new insights into the evolution of *B. melitensis* in the Mediterranean. We have confirmed that the Western Mediterranean lineage was present in the region since at least the 14^th^ century CE, and we have narrowed the estimated time-scale for the expansion of the West Mediterranean clade thanks to strong date priors using the ancient genome [26, 27]. More genomes—both ancient and modern—from a wider geographic range are required to understand the nuances of how *B. melitensis* evolved and was disseminated across the Mediterranean and eventually the globe.

## Materials and methods

### Ethics statement

Ethics approval was obtained from the Hamilton Integrated Research Ethics Board associated with McMaster University (Project Number: 13841-T). Dr. Gino Fornaciari was given written legal and ethical permission to study Sante Brancorsini by the Congregation of Saints of the Catholic Church in Rome.

### Context

The sample used for this study is a kidney stone obtained from the mummified remains of Sante Brancorsini, an Italian noble born in 1343 CE [74]. Brancorsini became a Franciscan friar after reportedly killing someone in self-defence at the age of twenty [74, 75]. He died in 1394 and was initially buried in a common grave on church grounds. The remains of Brancorsini were then moved to the convent wall in 1395 after a lily grew on his grave. Brancorsini was beatified in 1769 and his remains were then transferred below an altar in a chapel named in his honour. Dr. Gino Fornaciari examined and sampled the partially skeletonized remains of Brancorsini in 1994 [75].

### Sample processing

A calcified nodule weighing 129.1 mg identified as a kidney stone was sent to the McMaster Ancient DNA Centre (McMaster University, Hamilton, Canada) for aDNA analysis. The entire stone was sampled in clean-room facilities and underwent six rounds of demineralization and digestion [76]. The supernatant from rounds 3 and 4 underwent purification, extraction, and double-stranded library preparation following previously published aDNA procedures [77] with previously published modifications [78]. Two further modifications were made to the extraction protocols: a fifteen hour ligation period and MinElute purification instead of a bead-based method. The kidney stone was processed alongside extraction and library blanks. The libraries were then sequenced on an Illumina HiSeq 1500 platform with 90 bp paired-end reads at the Farncombe Family Digestive Health Research Institute. The library was further sequenced upon the discovery of *B. melitensis* during a preliminary metagenomic analysis. Additional information concerning the processing of the kidney stone can be found in the original study [16].

### Bioinformatics analysis

Adapter detection, read trimming, and merging were performed by fastp [79]. The data generated over multiple sequencing lanes and runs were pooled when applicable, and Kraken 2 [80] was used to determine the overall metagenomic composition of the samples. A standard Kraken 2 database with some slight modifications—a kmer size of 25bp and no minimizer—to account for the smaller read lengths of aDNA fragments was used [45]. The trimmed reads underwent string deduplication via prinseq [81] prior to the metagenomic analysis.

The reads were then mapped against the human genome—GRCh38 [82]—with BWA aln [83] using previously published parameters [84]. A minimum length of 30bp and mapping quality of 30 was required for a read to be successfully matched. Unsuccessful fragments were then separately mapped to the *B. melitensis* bv. 1 strain 16M (GCF_000007125) genome to confirm its presence and determine the authenticity of the reads. SPAdes 3.14.1 was used to *de novo* assemble the unsuccessful human fragments using kmer sizes of 15, 21, and 29 to account for the short read lengths [85]. Contigs smaller than 200bp were removed from the assembly with the rest being analyzed by BlastN [86] using the nt database to identify *B. melitensis* fragments. The blast output was analyzed using a previously published script and required that at least 50% of the identifications were of the same taxon [16]. The contigs were then analyzed with MOB-suite to identify any potential plasmids or mobile genetic elements [51]. A pan-genome was then created with 323 *B. melitensis* genomes obtained from NCBI on 2019–09-17 using NCBImeta [87] along with the ancient assembly. These sequences were annotated with Prokka [88] using proteins from GCF_000007125 as the initial standard. The resulting genome annotations were then compiled by Roary [60] to create the *B. melitensis* pan-genome and calculate the PAV matrix. See the supplemental information for the pan-genome mapping results (S1 Appendix)

Successfully mapped human and *B. melitensis* reads were deduplicated based on their coordinates with bam-rmdup (https://bitbucket.org/ustenzel/biohazard-tools/src/master/). mapDamage 2.0 [89] was used to estimate the level of deamination present across the mapped reads. Fragment length distributions (FLD) and mapping mismatches were also extracted from the mapped reads. The read depths were normalized based on the GC content of their respective genes to control for biases introduced during sequencing and DNA preservation [46, 90]. This was done by calculating the GC content over a sliding window and creating a linear regression in which the top 1% of residuals were filtered out. Once this was done, a correction factor was calculated by subtracting the mean of sequencing depths by the predicted value from the regression. This correction factor was then added to the original sequencing depth value [91].

### Phylogenetic analysis

A core SNP alignment of the 323 *B. melitensis* genomes from NCBI and the ancient *B. melitensis* assembly was created using Snippy (https://github.com/tseemann/snippy) with *B. abortus* 2308 (GCF_000054005) as the outgroup and *B. melitensis* 16M (GCF_000007125) as the reference strain. Recombinant regions were removed with Gubbins [93] and the resulting alignment used to create a maximum-likelihood (ML) phylogeny with IQ-TREE 2 [93]. The nucleotide substitution model (TVM+F+ASC+R5) was selected using ModelFinder [94] along with the added requirement that ascertainment bias correction was included. The initial phylogeny was then trimmed using Treemmer [95] down to a root-to-tip length (RTL) of 95% to remove redundant genomes. An additional eight genomes were removed due to long branches and unclear isolation dates. The remaining 127 genomes then underwent the same phylogenetic pipeline, with Gubbins removing 2485 bp (12.42% from the alignment) prior to creating the phylogeny. Sequences were labelled based on their ST which was identified using SRST2 [96] and the *Brucella* MLST panel from PubMLST [97].

After identifying that the ancient genome was part of the Western Mediterranean clade, a second set of phylogenies only comprising the relevant strains from this location were estimated and rooted using *B. abortus* 2308. The Gubbins analysis removed 582 bp (6.58%) of the core SNP alignment. While the same settings were used for this phylogeny, a different nucleotide substitution model (K3Pu+F+ASC) was selected with ModelFinder [94] where K3Pu is the unequal bases version of K81 [98].

To determine if the sequence data contained any temporal signature, sampling dates (or sequencing dates when the former was unavailable) were assigned to the pruned phylogenies and a Root-to-tip (RTT) regression was performed for the global and Western Mediterranean clade phylogenies using TempEst [52]. A least squares dating analysis with LSD2 was also used to confirm the temporal signal within the phylogenies through previously published settings [45, 99]. This was done by running LSD2 on the individual trees from each bootstrap and summarizing the results with treeannotator [100]. The heights of each node set by the mean of the bootstrap distribution. The resulting phylogeny was then used to plot the dated trees.

BEAST 1.10.4 (Prerelease) [100] was also used to estimate Bayesian phylogenies of the trimmed global and Western Mediterranean phylogenies. A strict molecular clock model along with a coalescent exponential demographic model was used to create the phylogeny, in line with previous studies [26, 27]. Chain lengths varying between 50 million and 1 billion generations with a 20% burn-in was used to ensure that the estimated sample sizes were above 200. While invariant sites were excluded from the core SNP alignment, the number of invariant sites were manually inserted into the XML files as a constant pattern to ensure that ascertainment bias did not affect the clock model. The Bayesian phylogenies were tested for a temporal signature using the BETS method [101] by comparing the marginal likelihood estimates of a heterochronous and isochronous phylogeny (S1 Appendix). To ensure that the log marginal likelihoods for the isochronous phylogenies could be calculated, the clock rate priors were deleted from the XML files.

### Functional analysis

A gene PAV analysis was performed using the matrix calculated by Roary [60]. A 99% core genome was calculated and all the genes were functionally annotated using the 2020 COG database [102]. Core genes absent from the ancient genome were annotated using the STRING database [55]. *Brucella* virulence factors [56] were also identified to determine if the relevant genes were present in the ancient genomes. This was done by both searching the gene names in the pan-genome as well as by identifying the COG functions of the ambiguous genes. A search for AMR genes in the ancient *B. melitensis* assembly was also performed using the Resistance Gene Identifier [57].

We performed a PAV analysis on the accessory genes of the *B. melitensis* pan-genome. The PAV metrics of the strains were transformed into a binary distance matrix and clustered using a PCoA. To ensure that redundant genes were not included an exclusion criterion was created: genes not present in at least five genomes were removed from the analysis. Following this, naive clustering of the *B. melitensis* genomes using their PCoA coordinates was done with the clara algorithm [103]. A silhouette plot [104] was used to determine the optimal number of clusters. A multiple correspondence analysis (MCA) plot was also created using the SNPs of accessory genes from modern and ancient genomes relative to the reference *B. melitensis* strain (GCF_000007125). Singleton SNPs were removed prior to creating the MCA as they would not provide any meaningful information.

## Supporting information

S1 Fig Kidney stone from Sante Brancorsini from which aDNA was obtained. The diameter of the stone is 0.5mm.
(TIFF)

S2 Fig Metagenomic profiles of currently known ancient samples. Metagenomic profiles were calculated at the genus taxonomic level. Numbers above each column indicate the total number of reads identified at the genus level or lower. The Geridu sample comes from a previously published ancient *B. melitensis* genome [13].
(TIF)

S3 Fig Global *B. melitensis* phylogeny. Core SNP Bayesian phylogeny of the global *B. melitensis* genome diversity. The red bars at the nodes indicate the 95% highest posterior density of the estimated date for a node whereas the text indicates the boundaries of the interval. The text along with coloured shaded regions indicate the estimated dates for sheep domestication and migration to specific regions [105]. When available, the genomes are labelled by the their country of isolation. *B. abortus* 2308 was the outgroup whereas *B. melitensis* 16M the reference genome.
(TIF)

S4 Fig Determining the temporal signal of the Global *B. melitensis* ML Phylogeny. Root-to-tip distances were calculated using TempEst [52] by selecting the best root using the heuristic residual mean squared option.
(TIF)

S5 Fig Maximum Likelihood Phylogeny the Western Mediterranean clade. Core SNP ML phylogeny of the Western Mediterranean phylogenetic group. The red bars at the nodes indicate the 95% highest posterior density of the estimated date for a node whereas the text indicates the boundaries of the interval. The text along with coloured shaded regions indicate the estimated dates for sheep domestication and migration to specific regions [105]. When available, the genomes are labelled by the their country of isolation. *B. abortus* 2308 was the outgroup whereas *B. melitensis* 16M the reference genome.
(TIF)

S6 Fig Determining the temporal signal of the Western Mediterranean ML phylogeny. Root-to-tip distances were calculated using TempEst [52] by selecting the best root using the heuristic residual mean squared option.
(TIF)

S7 Fig MCA of SNPs found in the accessory genome. SNPs were created using the VCF files from the core SNP alignment for the global phylogeny. Singletons were removed from the analysis prior to the MCA. **a)** contains the first two axes of the MCA whereas **b)** consists of the first and third.
(TIF)

S8 Fig Heatmap of PAV in the accessory genome of *B. melitensis*. Red indicates that a gene is present whereas blue is absent. Rows and columns were clustered using the Ward.D2 algorithm. The horizontal yellow lines represent the wayward ST 8 genomes whereas the vertical lines indicate genes that are uniquely present or absent in the Africa/America cluster.
(TIF)

S9 Fig Coverage plot of NC_00317.1 and NC_00318.1. Coverage plots for the first (NC_003317) and second (NC_003318) chromosomes in *B. melitensis* (GCF_000007125) for our **a)** ancient genome and **b)** a previously published [13] sample. A sliding window of 0.1% was used for illustrative purposes. The first track indicates the mean read depth with the red line representing the overall mean. The second track indicates the number of SNPs identified in the same window while the third is the GC content.
(TIF)

S10 Fig Heatmap of PAV in the Core Genome of *B. melitensis*. Red indicates that a gene is present whereas blue is absent. Rows and columns were clustered using the Ward.D2 algorithm.
(TIF)

S11 Fig Euler diagram of accessory genes in each PCoA cluster. Genes were identified as part of a cluster if it was present in more than genome. The counts indicate how many genes are found by the intersecting groups.
(TIF)

S12 Fig Maximum Likelihood Phylogeny of the Western Mediterranean clade with all known ancient genomes. (TIF)

S13 Fig Pan-genome mapping thresholds. **a)** Determining the number of additional genes identified at each CV threshold. The dashed red line indicates the chosen threshold (≤ 1.5) for the analysis. **b)** Distribution of mean gene coverages for the mapping of the ancient *B. melitensis* strain to the pan-genome. The dashed black line indicates the mean coverage while the blue and orange lines represent −2*σ* and −3*σ* from the mean.
(TIF)

S14 Fig Principal Coordinate Analyses (PCoA) of accessory gene PAV from the Mapping Methodology. Sequence types are identified using SRST2, although the outlier ST 8 genomes (from NIPH) are in black.
(TIF)

S1 File STRING results and missing core gene BLASTX results. NIPH Compared to Africa and America: Cells in green represent genes present in Africa/America Cluster, red is otherwise. High Coverage Contigs CNV: Cells in green represent accessions with more than two hits; red indicates accessions with only one hit. High Coverage Contigs RefSeq Protein: Yellow cells indicate cases where multiple definitions were identified. Accessory Genes by Cluster: Green indicates that a gene is present whereas red is absent. Yellow cells indicate cases where multiple definitions were identified.
(XLSX)

S1 Appendix Supplemental information. (PDF)
