# Supplementary figures and images for: A 14th century CE Brucella melitensis genome and the recent expansion of the Western Mediterranean clade
Source: PLoS Pathog. 2023 Jul 31;19(7):e1011538. doi: 10.1371/journal.ppat.1011538 (PMC10414615; doi:10.1371/journal.ppat.1011538)

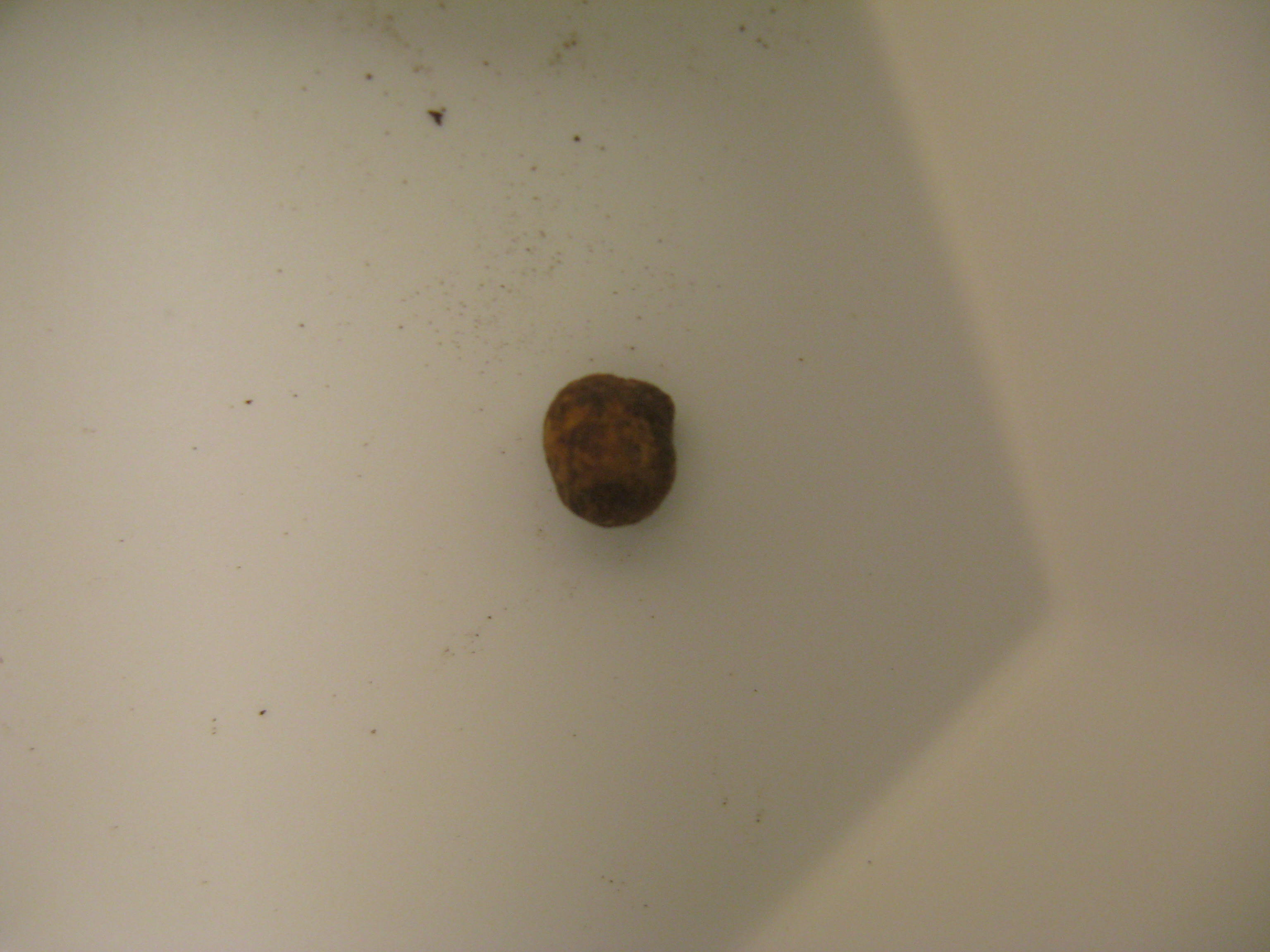

Supplement: S1 Fig — The diameter of the stone is 0.5mm. (TIFF) [file ppat.1011538.s001.tiff]

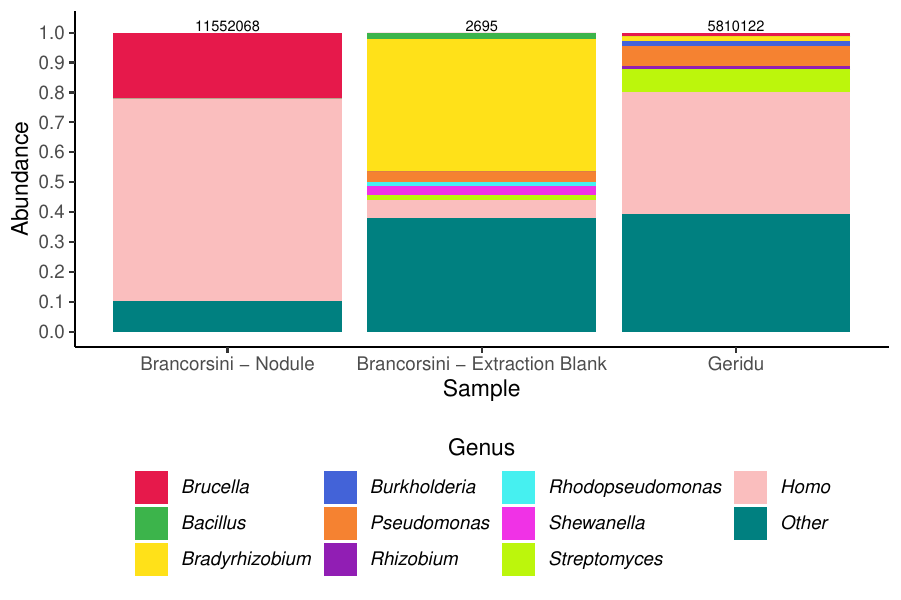

Supplement: S2 Fig — Metagenomic profiles were calculated at the genus taxonomic level. Numbers above each column indicate the total number of reads identified at the genus level or lower. The Geridu sample comes from a previously published ancient B. melitensis genome [13]. (TIF) [file ppat.1011538.s002.tif]

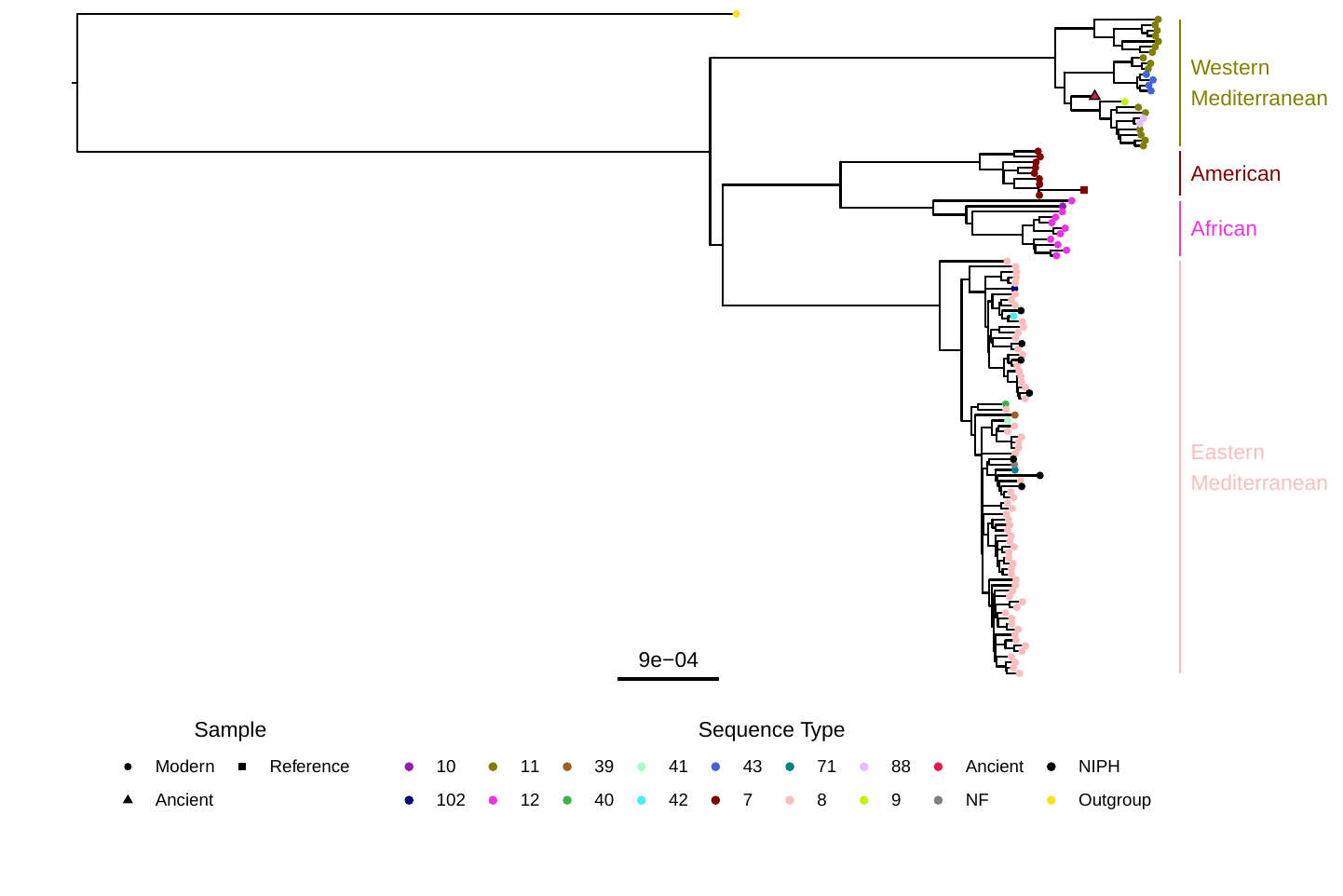

Supplement: S3 Fig — Core SNP Bayesian phylogeny of the global B. melitensis genome diversity. The red bars at the nodes indicate the 95% highest posterior density of the estimated date for a node whereas the text indicates the boundaries of the interval. The text along with coloured shaded regions indicate the estimated dates for sheep domestication and migration to specific regions [105]. When available, the genomes are labelled by the their country of isolation. B. abortus 2308 was the outgroup whereas B. melitensis 16M the reference genome. (TIF) [file ppat.1011538.s003.tif]

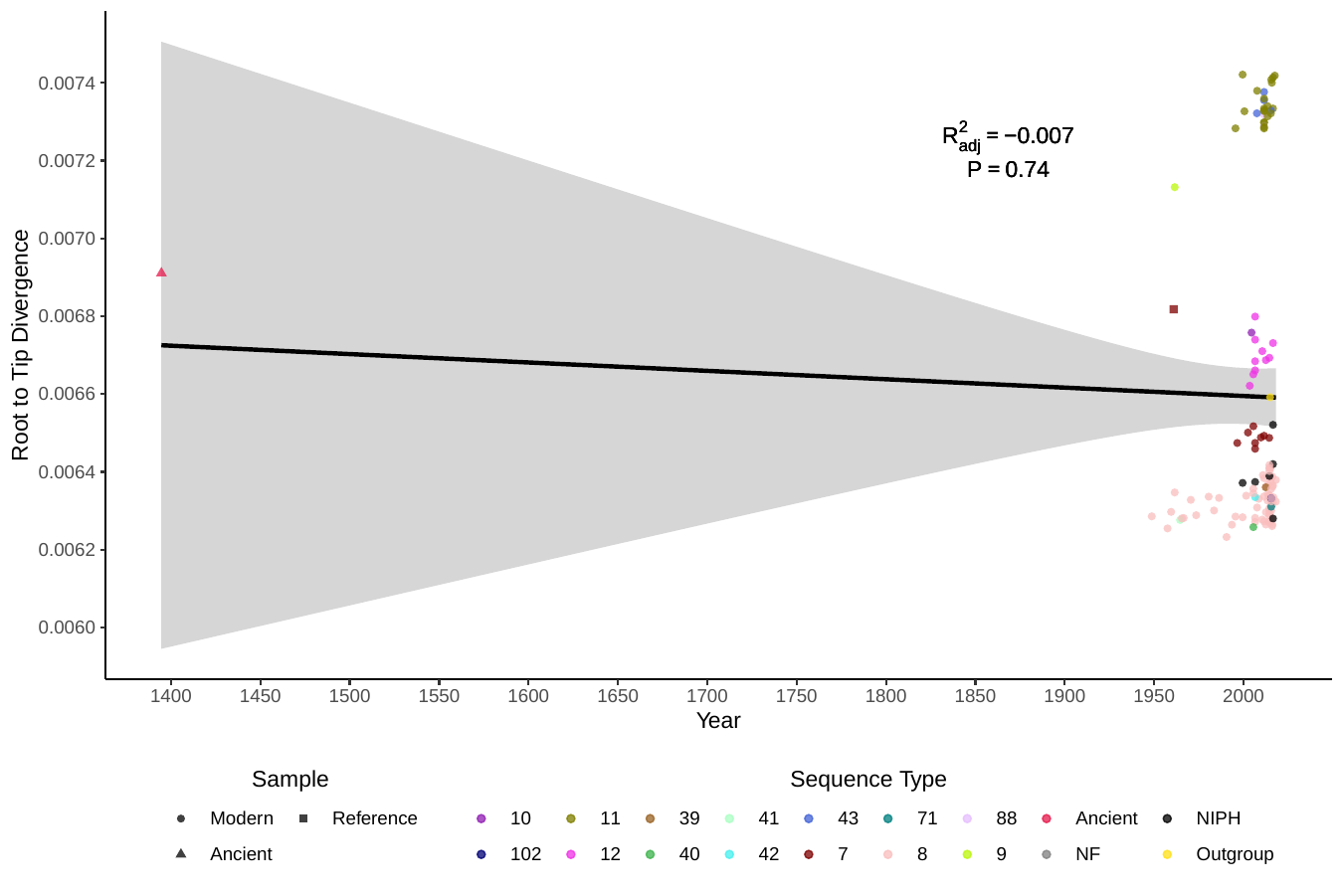

Supplement: S4 Fig — Root-to-tip distances were calculated using TempEst [52] by selecting the best root using the heuristic residual mean squared option. (TIF) [file ppat.1011538.s004.tif]

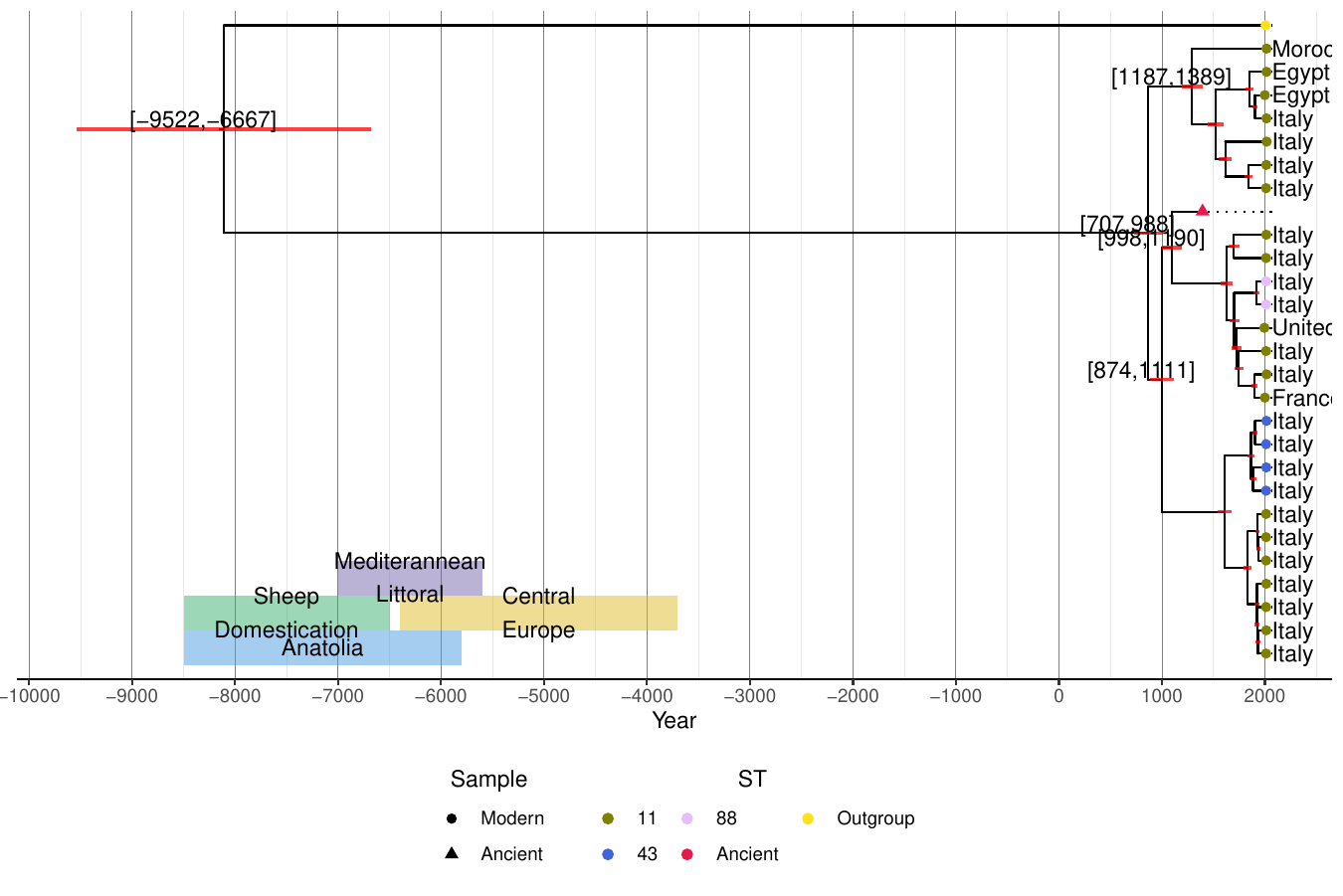

Supplement: S5 Fig — Core SNP ML phylogeny of the Western Mediterranean phylogenetic group. The red bars at the nodes indicate the 95% highest posterior density of the estimated date for a node whereas the text indicates the boundaries of the interval. The text along with coloured shaded regions indicate the estimated dates for sheep domestication and migration to specific regions [105]. When available, the genomes are labelled by the their country of isolation. B. abortus 2308 was the outgroup whereas B. melitensis 16M the reference genome. (TIF) [file ppat.1011538.s005.tif]

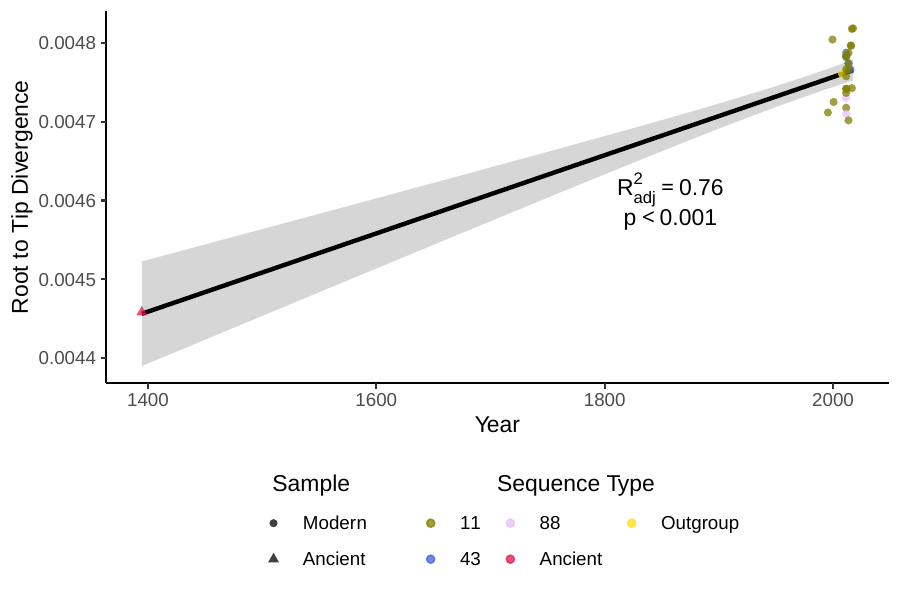

Supplement: S6 Fig — Root-to-tip distances were calculated using TempEst [52] by selecting the best root using the heuristic residual mean squared option. (TIF) [file ppat.1011538.s006.tif]

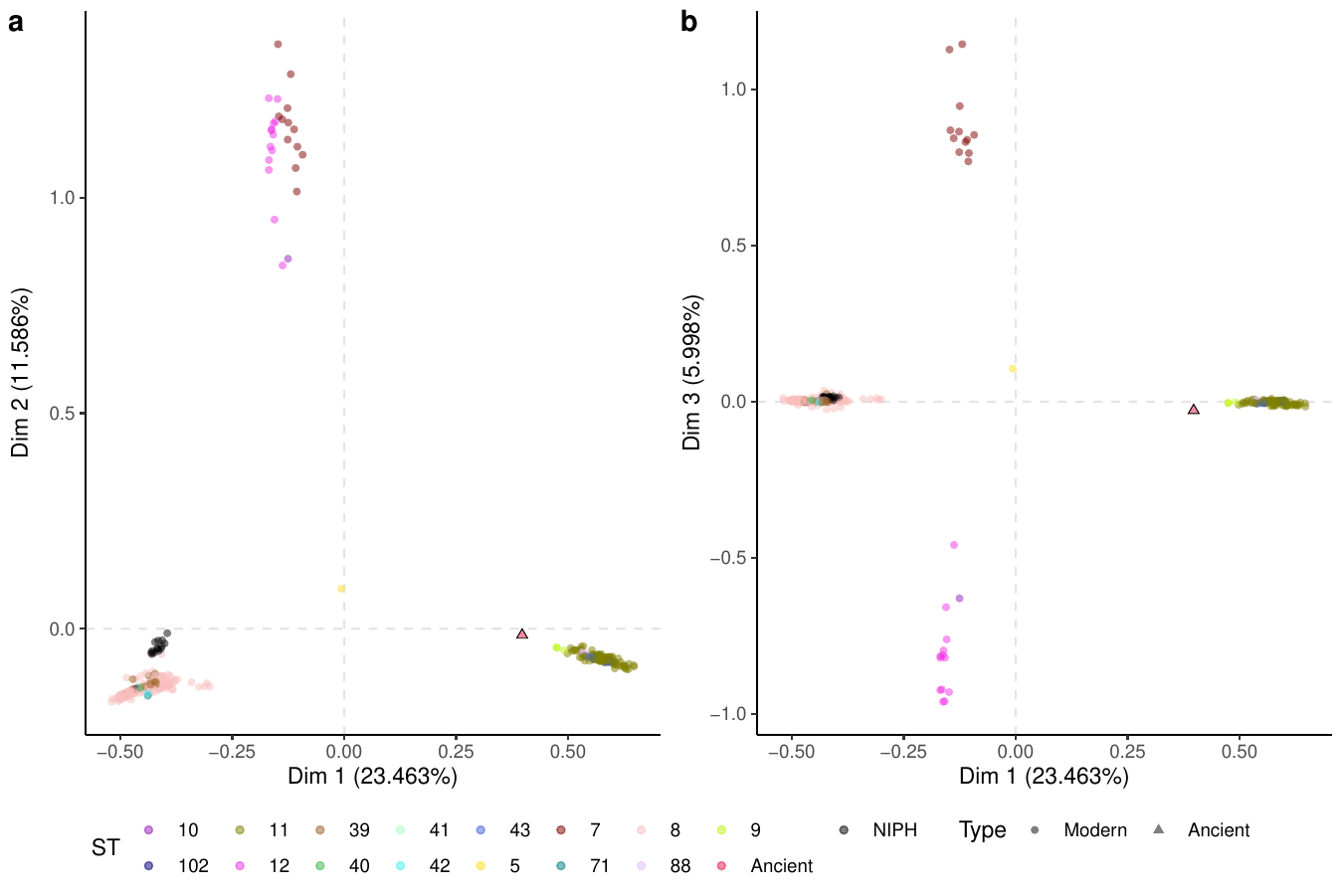

Supplement: S7 Fig — SNPs were created using the VCF files from the core SNP alignment for the global phylogeny. Singletons were removed from the analysis prior to the MCA. a) contains the first two axes of the MCA whereas b) consists of the first and third. (TIF) [file ppat.1011538.s007.tif]

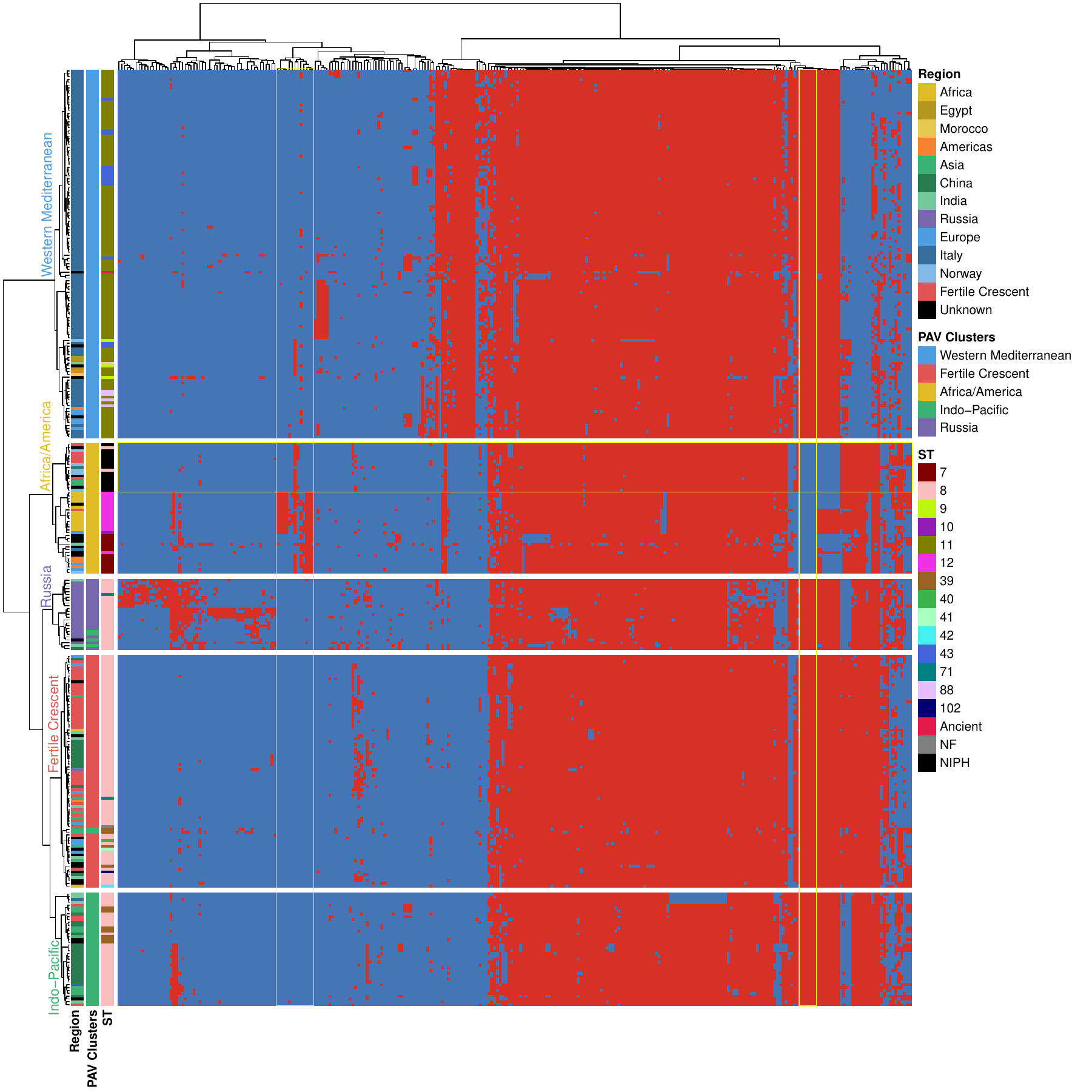

Supplement: S8 Fig — Red indicates that a gene is present whereas blue is absent. Rows and columns were clustered using the Ward.D2 algorithm. The horizontal yellow lines represent the wayward ST 8 genomes whereas the vertical lines indicate genes that are uniquely present or absent in the Africa/America cluster. (TIF) [file ppat.1011538.s008.tif]

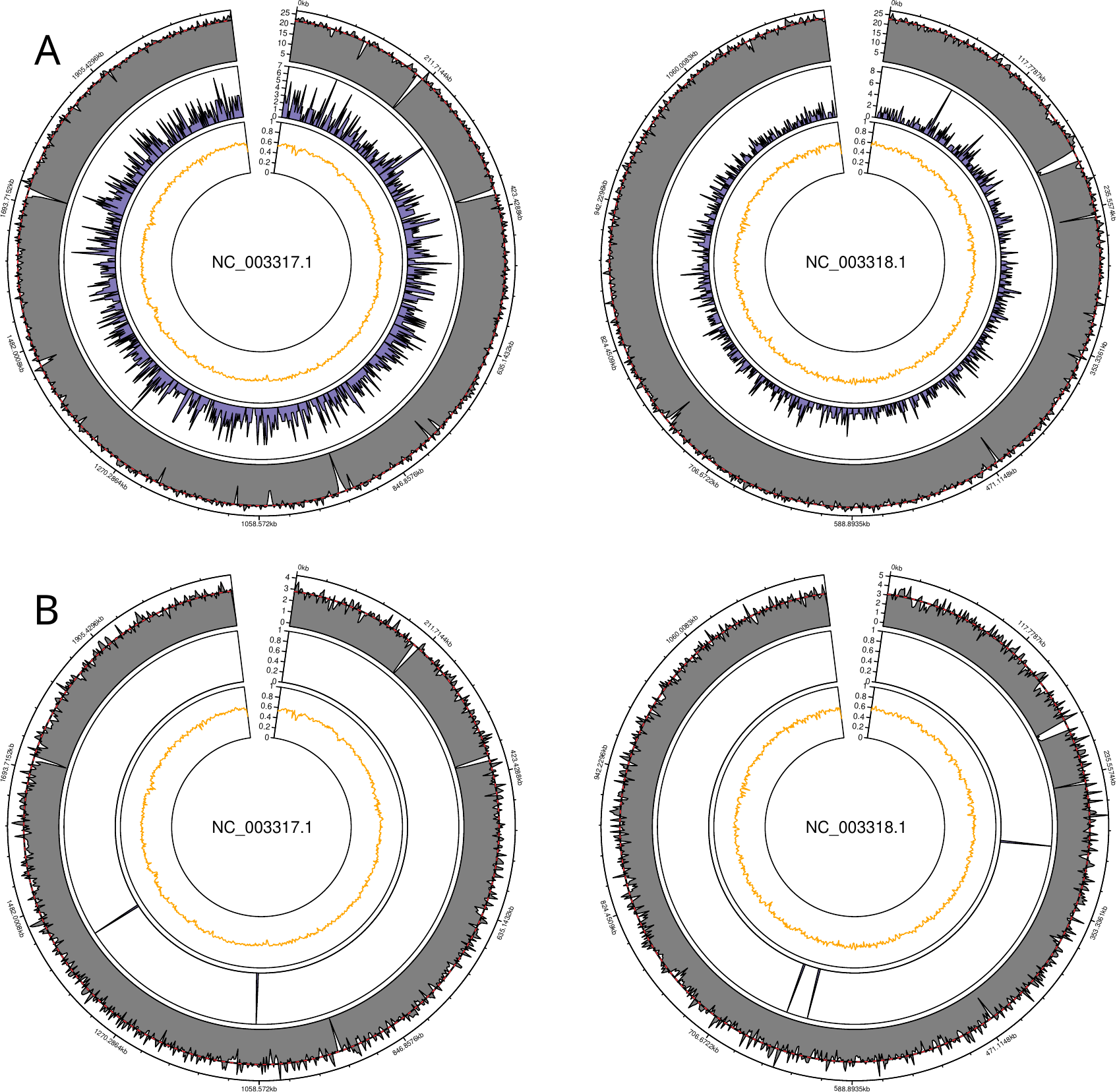

Supplement: S9 Fig — Coverage plots for the first (NC_003317) and second (NC_003318) chromosomes in B. melitensis (GCF_000007125) for our a) ancient genome and b) a previously published [13] sample. A sliding window of 0.1% was used for illustrative purposes. The first track indicates the mean read depth with the red line representing the overall mean. The second track indicates the number of SNPs identified in the same window while the third is the GC content. (TIF) [file ppat.1011538.s009.tif]

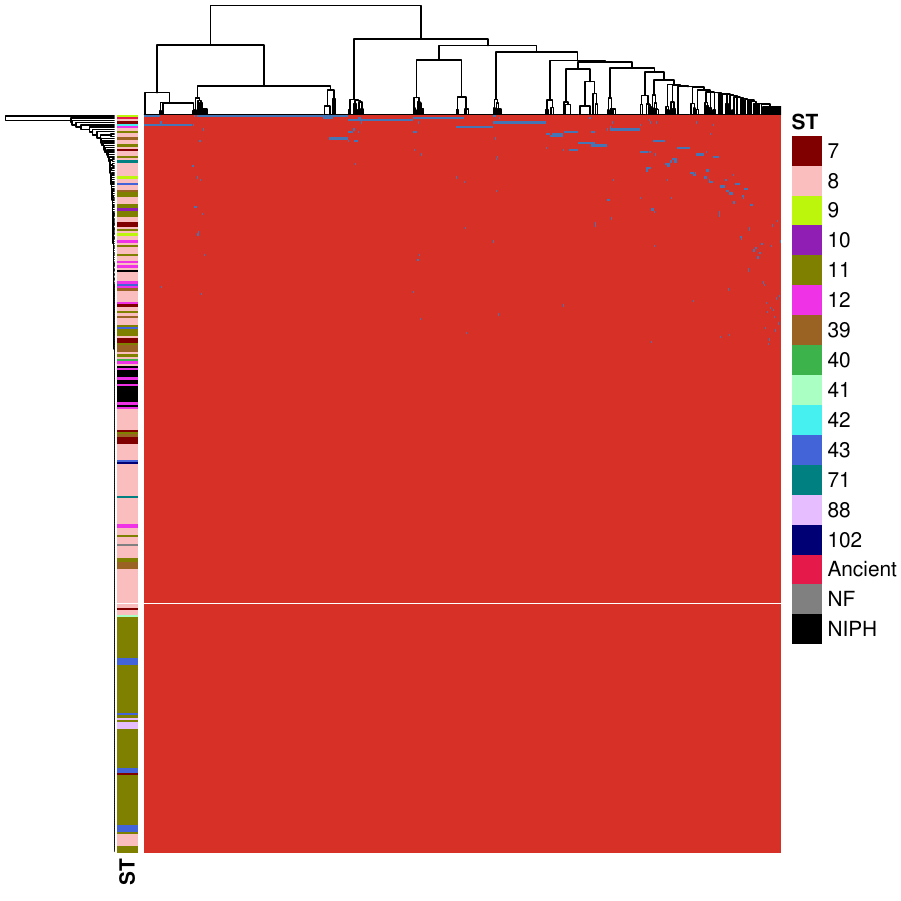

Supplement: S10 Fig — Red indicates that a gene is present whereas blue is absent. Rows and columns were clustered using the Ward.D2 algorithm. (TIF) [file ppat.1011538.s010.tif]

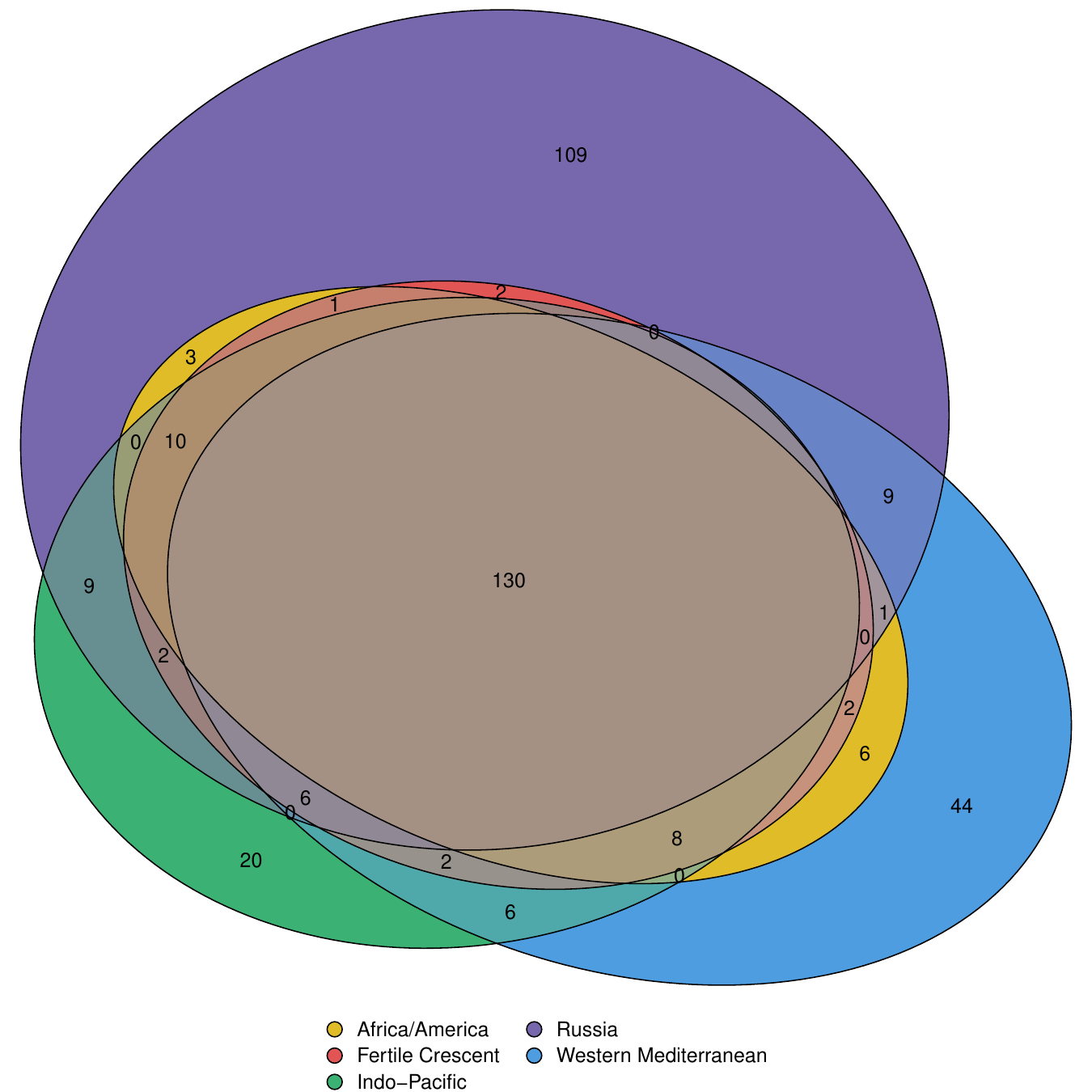

Supplement: S11 Fig — Genes were identified as part of a cluster if it was present in more than genome. The counts indicate how many genes are found by the intersecting groups. (TIF) [file ppat.1011538.s011.tif]

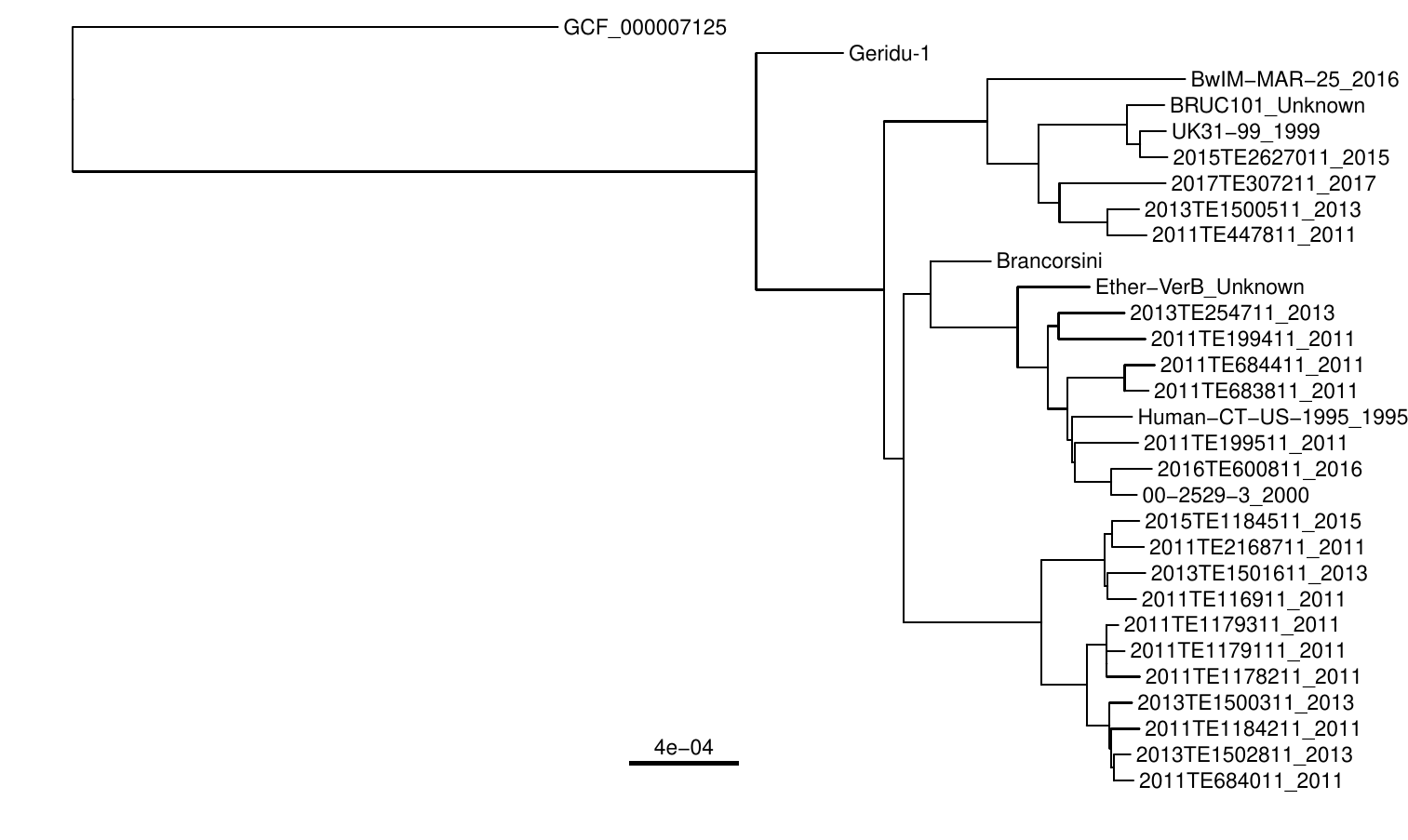

Supplement: S12 Fig — (TIF) [file ppat.1011538.s012.tif]

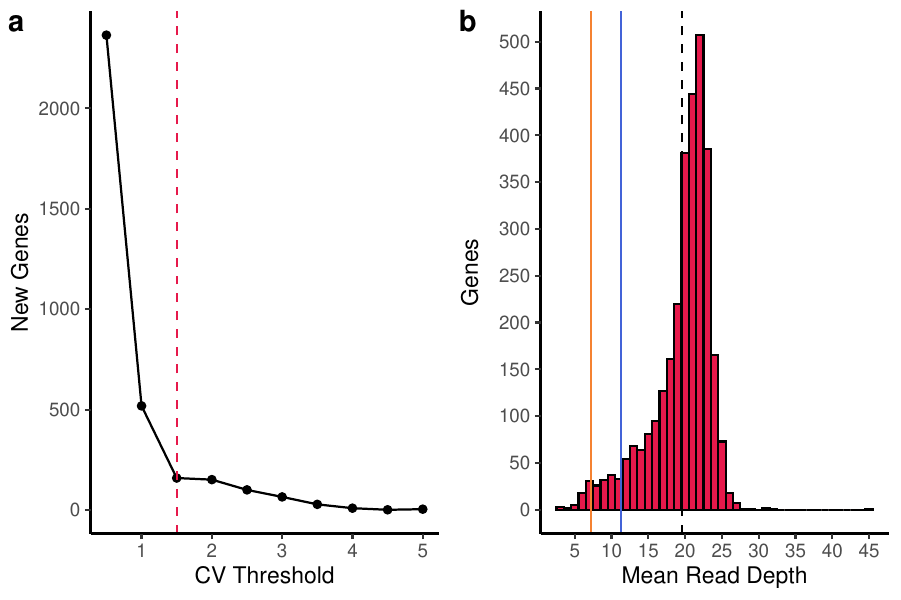

Supplement: S13 Fig — a) Determining the number of additional genes identified at each CV threshold. The dashed red line indicates the chosen threshold (≤ 1.5) for the analysis. b) Distribution of mean gene coverages for the mapping of the ancient B. melitensis strain to the pan-genome. The dashed black line indicates the mean coverage while the blue and orange lines represent −2σ and −3σ from the mean. (TIF) [file ppat.1011538.s013.tif]

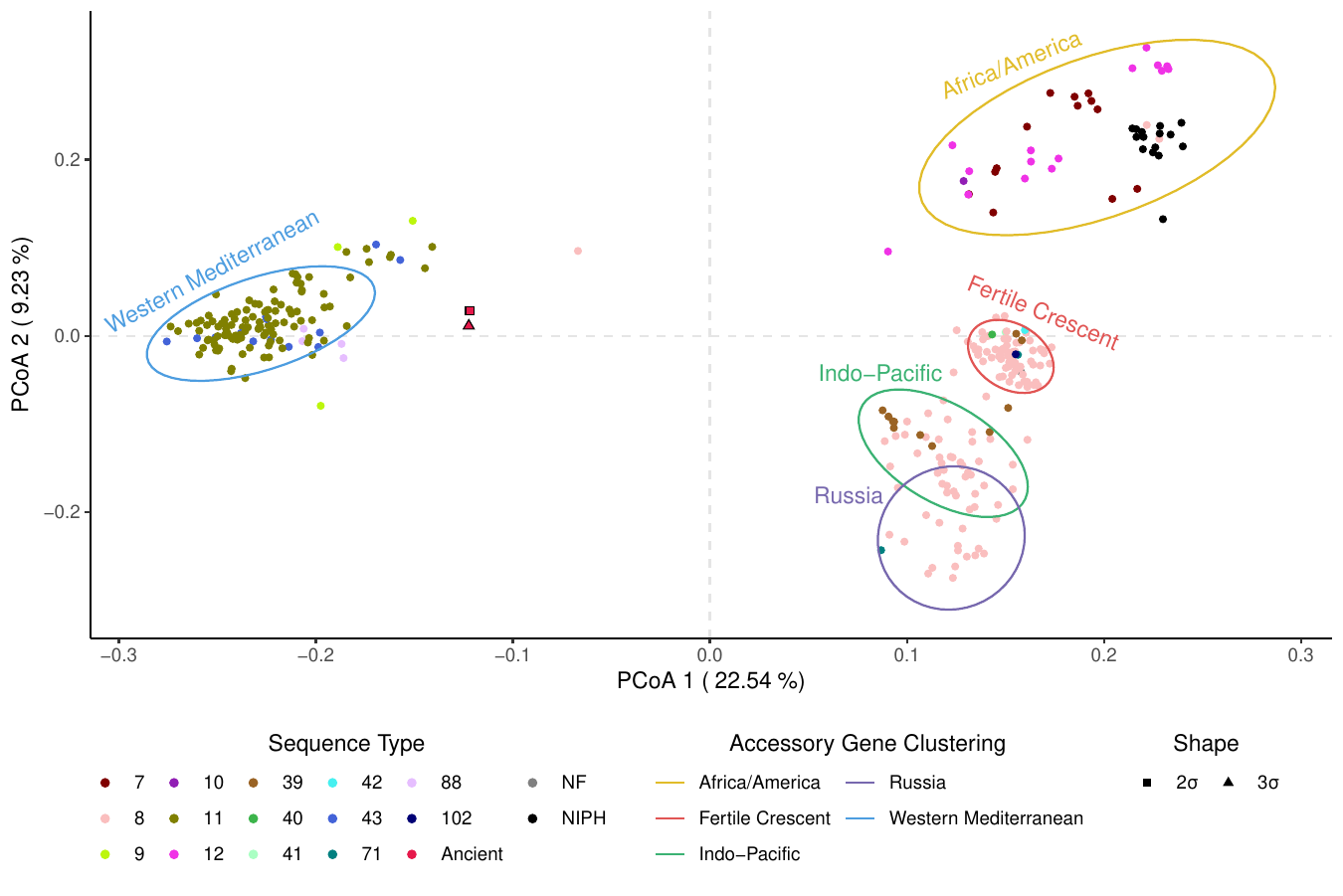

Supplement: S14 Fig — Sequence types are identified using SRST2, although the outlier ST 8 genomes (from NIPH) are in black. (TIF) [file ppat.1011538.s014.tif]
