# Supplementary material for: A 14th century CE Brucella melitensis genome and the recent expansion of the Western Mediterranean clade
Source: PLoS Pathog. 2023 Jul 31;19(7):e1011538. doi: 10.1371/journal.ppat.1011538 (PMC10414615; doi:10.1371/journal.ppat.1011538)
Supplement: S1 Appendix — (PDF) [file ppat.1011538.s016.pdf]

Supplementary Information for: A 14th century CE *Brucella melitensis* genome and its recent Western Mediterranean expansion

George S. Long[1,2*◕], Jessica Hider[2,3*◕], Ana T. Duggan[2,3], Jennifer Klunk[1,2,4], Katherine Eaton[2,3], Emil Karpinski[1,2], Valentina Giuffra[5], Luca Ventura[6,7], Tracy L. Prowse[3], Antonio Fornaciari[5], Gino Fornaciari[8], Edward C. Holmes[9], G. Brian Golding[1], Hendrik N. Poinar[2,3,10,11,12*]

July 12, 2023

1 Department of Biology, McMaster University, Hamilton, Canada
2 McMaster Ancient DNA Centre, Departments of Anthropology and Biochemistry, McMaster University, Hamilton, Canada
3 Department of Anthropology, McMaster University, Hamilton, Canada
4 Daicel Arbor Biosciences, Ann Arbor, Michigan, United States of America
5 Division of Paleopathology, Department of Translational Research and New Technologies in Medicine and Surgery, University of Pisa, Pisa, Italy
6 Department of Biotechnological and Applied Clinical Sciences, University of L'Aquila, L'Aquila, Italy
7 Division of Pathology, San Salvatore Hospital, Coppito, Italy
8 Maria Luisa di Borbone Academy, Viareggio, Italy
9 Sydney Institute for Infectious Diseases, School of Medical Sciences, University of Sydney, Sydney, Australia
10 Department of Biochemistry, McMaster University, Hamilton, Canada
11 Michael G. DeGroote Institute for Infectious Disease Research, McMaster University, Hamilton, Canada
12 CIFAR Humans and the Microbiome Program, Toronto, Canada

◕These authors contributed equally to this work.
longg2@mcmaster.ca, hiderjessica@gmail.com, and poinarh@mcmaster.ca

Contents

1 Supplemental Introduction **3**

2 Supplemental Materials & Methods **4**
2.1 Sample Processing 4
2.2 Pan-genome Analysis 4

3 Supplemental Results **4**
3.1 Geridu Exclusion 4
3.2 Pan-genome Mapping Results 5

4 Tables **9**
4.1 Tables 9

1 Supplemental Introduction

There are several lineages of *Brucella melitensis* circulating in the modern Mediterranean region [1, 2, 3, 4]. The diversity of these lineages has led some researchers to hypothesize that *B. melitensis* either originated or was highly endemic in the Mediterranean for an extended period, especially in Italy [1, 4]. This has raised questions concerning how the current phylogeographic distribution of *B. melitensis* could be explained by historical human activities. The widespread movement of infected animals, their products, and other contaminated goods has been suggested as a possible reason for the current distribution and diversity of *B. melitensis* in the Mediterranean. Its main hosts are sheep and goats, which are easy to move and trade due to their small size and low cost [1, 4].

MLVA and MLSA have identified similar phylogeny topology to SNP methods, with 3 main lineages, West Mediterranean, East Mediterranean, and an Americas lineage [2, 5, 4, 6, 7, 8]. These lineages show spatial clustering in certain regions of the world. To understand the real and perceived geographic clustering of these lineages it is important to look at their origin. These lineage-region assignments originate from MLVA studies with the reference genomes of the three biovars for *B. melitensis* and their region of origin as well as the origins of other genomes falling into the clades associated with the biovars [6, 7]. The *B. melitensis* Ether strain (biovar 3) was isolated in Italy, all related strains were deemed Western Mediterranean (Genotype I with SNP data). The *B. melitensis* 63/9 (biovar 2) was isolated in China and was designated East Mediterranean (Genotype II with SNP data), *B. melitensis* 16M (biovar 1) was isolated in the United States and it and related strains form the Americas lineage (Genotype V). Notably the original data was not discrete in terms of geography, for example, the West Mediterranean lineage contained seven genomes from the Switzerland out of 27 total [7]. The addition of more genomes and phylogenetic studies has also revealed that some geographic associations of lineages are tenuous. For example, the East Mediterranean lineage is now known to have the largest geographic range, including Europe, the Middle East, and Asia [4, 3, 9].

Some SNP studies also separate out two other groupings and link them with geographic regions. Genotype III was designated as the African clade because five of six genomes stemmed from Africa [4] and is supported by other SNP studies [10, 2, 3] as well as MLVA[11]. Although it was recommended that recommend the clade not be thought of as a geographic grouping, noting that some African genomes (e.g., Algeria) are part of the West Mediterranean clade and suggest a connection with Italy (Lounes et al., 2014, Foster 2018). Some studies also identify another group associated with the genome *B. melitensis* B115 and designate it as Genotype IV (European lineage, sometimes called Malta-Portugal [3, 4], while more recent work places the Malta-Portugal group within the American lineage [2]. MLSA supports the placement of these African genomes with the American lineage [9]. This makes sense as SNP analyses have identified genotypes IV and V as emerging from a common ancestor derived from Genotype II [4, 8].

Our focus is primarily on the Western Mediterranean clade because this lineage predominantly includes genomes isolated in Italy [1, 2, 9] and the genome we have recovered is from this region as well as the only other ancient genome [5]. Other countries that have isolates in the West Mediterranean clade include Algeria, Tunisia, Morocco, Spain, France, Germany, Switzerland, and Egypt [4, 2, 12, 9]. Though other lineages are found in the Mediterranean (East Mediterranean and European/Malta-Portugal), the West Mediterranean lineage occupies the most basal node on the tree [2, 3, 4]. It is argued that the prevalence of it in the region indicates a long-standing presence and possibly that it originated in the Mediterranean [1, 2, 4]. This lineage is also the most common in Italy, for example MLVA has identified 96.5% of Italian isolates as West Mediterranean and in SNP-based work all Italian genomes were West Mediterranean [1, 2].

Regarding the evolution of the West Mediterranean lineage, previous work has identified that the diversification of the lineage began approximately 4500 BCE [3] while other work has identified a divergence date of 3500 [5768.6, 1746.9] BCE [2]. Domesticated sheep and goats were in the Central Mediterranean by this time and subsequent domestication and expansion events were also occurring or soon to occur (e.g., wool sheep found in Italy 4000-2000 BCE) [13, 14, 2] and it is therefore possible that *B. melitensis* arrived with infected ruminants stemming from the Fertile Crescent. Subsequently the Western Mediterranean lineage likely evolved in this region [2]. The ancient Sardinian genome, Geridu-1, was found to be part of The West Mediterranean lineage [5]. This provides further support for its historic presence in the Mediterranean, and more specifically Italy.

2 Supplemental Materials & Methods

2.1 Sample Processing

A kidney stone was sent to the McMaster Ancient DNA Centre for aDNA analysis as it was thought to be caused by renal tuberculosis. It was identified as a kidney stone from its appearance and location in the pelvic region (Fornaciari, personal communication).

The entire kidney stone (129.1 mg) was sampled in the cleanroom facility of the McMaster Ancient DNA Centre. Demineralization and digestion was performed [15], the process (EDTA incubation, centrifugation, removal of supernatant; and digestion buffer incubation, centrifugation, removal of supernatant) was repeated six times to ensure the nodule was substantially digested.

Silica-based extraction and column purification was completed on a combination of the supernatant from rounds 3 and 4 of demineralization and digestion steps using a modified version of a previously published methodology [16, 17]. Double-stranded library preparation was performed [18, 19], with a modification to a fifteen-hour ligation and MinElute PCR purification (Qiagen) instead of SPRI bead clean-up between steps. The kidney stone was processed alongside extraction and library blanks. The DNA libraries were pooled and gel excised to $\sim 150 - 500$ bp in length. The pooled libraries were shotgun sequenced at the Farncombe Family Digestive Health Research Institute (McMaster University, Hamilton, Canada) on partial lanes of the Illumina HiSeq 1500 platform using 90 bp paired end reads. The library was sequenced to greater depth on an additional sequencing run after the discovery of *B. melitensis* via metagenomic analysis. Further information can be found in the original study [20].

2.2 Pan-genome Analysis

A pan-genome mapping approach to characterize the ancient genome was also performed following a previously developed method [21]. The difference in the mapping approach is that the filtered reads were mapped against a pan-genome created from the 323 modern *B. melitensis* strains. The read depths were then normalized based on the GC content of their respective genes. Following this, the normalized read depths of the ancient *B. melitensis* pan-genome mapping were converted into a presence absence variation (PAV) metric [21]. A coefficient of variation (CV) threshold of ≤ 1.5 was used to filter genes which contained large regions of stacked reads. This threshold was determined by identifying the CV value (in increments of 0.5) which had the largest decrease in new genes. Two different thresholds for the mean read depth – $\bar{Depth}_{Genes} - 2\sigma$ and $\bar{Depth}_{Genes} - 3\sigma$ – were selected to ensure that only genes with sufficient coverage were selected.

3 Supplemental Results

3.1 Geridu Exclusion

Attempts were made to include the previously published *B. melitensis* genome (Geridu-1) [5] in the main manuscript, however, replication and coverage issues precluded it from our analysis. The results from our analysis of this first ancient genome are included below.

The authentication results from the Geridu-1 data signalled potential issues. While evidence of deamination is present and comparable to the *B. melitensis* results from the Brancorsini data, this is only the case at the $3'$ end of the fragments. Only a negligible amount of deamination is present at the $5'$ end. This contradicts its previously published results where damage was present at both ends of the molecule[5], something which we were not able to replicate. The FLDs, however, closely resembles the previously published data and are substantially different from the Brancorsini results. The FLDs are most likely due to the atypical laboratory methods of the original study[5]. The edit distance results indicate that the proper references were used during mapping.

The pan-genome mapping results for the Geridu-1 library were substantially lower than what was achieved with our genome at $2.364\ [2.334, 2.394]\times$ while also identifying 2805 genes for coverage results against *B. melitensis* 16M). GC bias correction also lead to the net loss of eleven genes, yet, the mean still did not significantly differ from the original results. A SNP analysis was not performed due to its low coverage. An analysis of its accessory genome presence/absence variation places the strain in the Western Mediterranean phylogenetic group.

A phylogenetic analysis including Geridu-1 places it basal to the Western Mediterranean phylogeny (Supplemental Figure S10). This recapitulates the broad results from the original study [5]. The issue here, however, is that its low coverage severely reduced the number of core SNPs available for the phylogeny (2373 core SNPs compared to 9484 for the Western Mediterranean phylogeny). Thus, the Geridu-1 genome was excluded from the phylogeny to ensure that most of the information possible would be used for the temporal dating.

3.2 Pan-genome Mapping Results

A pan-genome mapping approach [21] was initially followed using a CV threshold of ≤ 1.5 (Fig S13a) and two mean gene coverage thresholds (-2σ and -3σ from the mean) to test the sensitivity of the method (Fig S13b). The broader of the two methods identified 2995 genes with a mean read depth of 19.80 $[19.93, 19.66]\times$ whereas the more conservative approach identified 2864 genes at a coverage of 20.28 $[20.16, 20.39]\times$ (Table S3). While over 90% of the core genome was identified in all the methods tested the *denovo* assembly contained a substantially greater number. This is likely not due to the number of identified genes, instead, it is a result of the underlying methods for gene identification.

Genes were identified in the *denovo* assembly method using `Prokka` to annotate contigs $\geq$ 200bp long. `Prokka` itself is not a feature prediction tool as it relies on specialized programs (`Prodigal, RNAmmer, Aragorn, SignalP,` and `Infernal` [22, 23, 24, 25, 26]) to find putative genes. `Roary` is then used to cluster genes and collate the results into a pan-genome [27]. It is `Roary` which provides the gene presence-absence table used to calculate the core genome and the functional analyses. This final step of the assembly analysis will also cluster similar genes into a single entry. Thus, the *denovo* assembly undergoes a thorough gene detection pipeline which is commonly used with modern genomes.

In contrast, the mapping method is reliant on the selection of modern strains and ambiguous coverage thresholds. In the case of the former, the criteria used to collect the modern strains will invariably lead to potential reference biases as certain genomes will be excluded for a variety of reasons. This bias, which is reduced due to the clonal nature of *B. melitensis* and its closed genome [28, 29, 30], can lead to an incomplete genetic diversity of the strain of interest. The lacking diversity can potentially lead to missing genes in pan-genome, which is especially pertinent in this study given that *B. melitensis* is undergoing purifying selection [28]. The biases introduced by the selection used for the modern genome selection is exacerbated by the mean gene depth and CV thresholds for the PAV analyses. While a more conservative threshold (represented by -2σ in Table S3) will ensure that the detected genes are truly present, there is a risk of a substantial number of false negatives. The lower threshold (-3σ) represents the differences between the two thresholds as it contains 131 more genes, however, it is still lacking several core elements. Annotating a *denovo* assembled ancient genome precludes these issues and ensures that the only bias introduced into the PAV analysis is the sequencing depth and preservation of the sample.

Despite the differences between the two methods, the PAV analysis results do not differ. The accessory PCoA still indicates that the ancient genome is part of the Western Mediterranean phylogenetic group with some minor differences.

References

1. Garofolo G, DiGiannatale E, DeMassis F, Zilli K, Ancora M, Camma C, Calistri P, and Foster J. Investigating genetic diversity of *Brucella abortus* and *Brucella melitensis* in Italy with MLVA-16. Infect Genet Evol 2013; 19:59–70. DOI: 10.1016/j.meegid.2013.06.021. Available from: https://www.ncbi.nlm.nih.gov/entrez/query.fcgi?db=PubMed&cmd=Retrieve&dopt=Citation&list_uids=23831636
2. Janowicz A, DeMassis F, Zilli K, Ancora M, Tittarelli M, Sacchini F, DiGiannatale E, Sahl J, Foster J, and Garofolo G. Evolutionary history and current distribution of the West Mediterranean lineage of *Brucella melitensis* in Italy. Microb Genom 2020; 6. DOI: 10.1099/mgen.0.000446. Available from: https://www.ncbi.nlm.nih.gov/entrez/query.fcgi?db=PubMed&cmd=Retrieve&dopt=Citation&list_uids=33030422
3. Pisarenko S, Kovalev D, Volynkina A, Ponomarenko D, Rusanova D, Zharinova N, Khachaturova A, Tokareva L, Khvoynova I, and Kulichenko A. Global evolution and phylogeography of *Brucella melitensis* strains. BMC Genomics 2018; 19:353. DOI: 10.1186/s12864-018-4762-2. Available from: https://www.ncbi.nlm.nih.gov/entrez/query.fcgi?db=PubMed&cmd=Retrieve&dopt=Citation&list_uids=29747573
4. Tan K, Tan Y, Chang L, Lee K, Nore S, Yee W, MatIsa M, Jafar F, Hoh C, and AbuBakar S. Full genome SNP-based phylogenetic analysis reveals the origin and global spread of *Brucella melitensis*. BMC Genomics 2015; 16:93. DOI: 10.1186/s12864-015-1294-x. Available from: https://pubmed.ncbi.nlm.nih.gov/25888205/
5. Kay G, Sergeant M, Giuffra V, Bandiera P, Milanese M, Bramanti B, Bianucci R, and Pallen M. Recovery of a medieval *Brucella melitensis* genome using shotgun metagenomics. mBio 2014; 5:e01337–14. DOI: 10.1128/mBio.01337-14. Available from: https://www.ncbi.nlm.nih.gov/entrez/query.fcgi?db=PubMed&cmd=Retrieve&dopt=Citation&list_uids=25028426
6. Whatmore A and Foster J. Emerging diversity and ongoing expansion of the genus *Brucella*. Infect Genet Evol 2021; 92:104865. DOI: 10.1016/j.meegid.2021.104865. Available from: https://www.ncbi.nlm.nih.gov/entrez/query.fcgi?db=PubMed&cmd=Retrieve&dopt=Citation&list_uids=33872784
7. AlDahouk S, Fleche P, Nockler K, Jacques I, Grayon M, Scholz H, Tomaso H, Vergnaud G, and Neubauer H. Evaluation of *Brucella* MLVA typing for human brucellosis. J Microbiol Methods 2007; 69:137–45. DOI: 10.1016/j.mimet.2006.12.015. Available from: https://www.ncbi.nlm.nih.gov/entrez/query.fcgi?db=PubMed&cmd=Retrieve&dopt=Citation&list_uids=17261338
8. Pelerito A, Nunes A, Nuncio M, and Gomes J. Genome-scale approach to study the genetic relatedness among *Brucella melitensis* strains. PLoS One 2020; 15:e0229863. DOI: 10.1371/journal.pone.0229863. Available from: https://www.ncbi.nlm.nih.gov/entrez/query.fcgi?db=PubMed&cmd=Retrieve&dopt=Citation&list_uids=32150564
9. Whatmore A, Koylass M, Muchowski J, Edwards-Smallbone J, Gopaul K, and Perrett L. Extended Multilocus Sequence Analysis to Describe the Global Population Structure of the Genus *Brucella*: Phylogeography and Relationship to Biovars. Front Microbiol 2016; 7:2049. DOI: 10.3389/fmicb.2016.02049. Available from: https://www.ncbi.nlm.nih.gov/entrez/query.fcgi?db=PubMed&cmd=Retrieve&dopt=Citation&list_uids=28066370
10. Georgi E, Walter M, Pfalzgraf M, Northoff B, Holdt L, Scholz H, Zoeller L, Zange S, and Antwerpen M. Whole genome sequencing of *Brucella melitensis* isolated from 57 patients in Germany reveals high diversity in strains from Middle East. PLoS One 2017; 12:e0175425. DOI: 10.1371/journal.pone.0175425. Available from: https://www.ncbi.nlm.nih.gov/entrez/query.fcgi?db=PubMed&cmd=Retrieve&dopt=Citation&list_uids=28388689
11. Foster J, Walker F, Rannals B, Hussain M, Drees K, Tiller R, Hoffmaster A, Al-Rawahi A, Keim P, and Saqib M. African Lineage *Brucella melitensis* Isolates from Omani Livestock. Front Microbiol 2017; 8:2702. DOI: 10.3389/fmicb.2017.02702. Available from: https://www.ncbi.nlm.nih.gov/entrez/query.fcgi?db=PubMed&cmd=Retrieve&dopt=Citation&list_uids=29379492
12. Lounes N, Cherfa M, LeCarrou G, Bouyoucef A, Jay M, Garin-Bastuji B, and Mick V. Human brucellosis in Maghreb: existence of a lineage related to socio-historical connections with Europe. PLoS One 2014; 9:e115319. DOI: 10.1371/journal.pone.0115319. Available from: https://www.ncbi.nlm.nih.gov/entrez/query.fcgi?db=PubMed&cmd=Retrieve&dopt=Citation&list_uids=25517901
13. Ciani E, Mastrangelo S, DaSilva A, Marroni F, Ferencakovic M, Ajmone-Marsan P, Baird H, Barbato M, Colli L, Delvento C, Dovenski T, Gorjanc G, Hall S, Hoda A, Li M, Markovic B, McEwan J, Moradi M, Ruiz-Larranaga O, Ruzic-Muslic D, Salamon D, Simcic M, Stepanek O, Curik I, Cubric-Curik V, and Lenstra J. On the origin of European sheep as revealed by the diversity of the Balkan breeds and by optimizing population-genetic analysis tools. Genet Sel Evol 2020; 52:25. DOI: 10.1186/s12711-020-00545-7. Available from: https://www.ncbi.nlm.nih.gov/entrez/query.fcgi?db=PubMed&cmd=Retrieve&dopt=Citation&list_uids=32408891

14. Deng J, Xie X, Wang D, Zhao C, Lv F, Li X, Yang J, Yu J, Shen M, Gao L, Yang J, Liu M, Li W, Wang Y, Wang F, Li J, Hehua E, Liu Y, Shen Z, Ren Y, Liu G, Chen Z, Gorkhali N, Rushdi H, Salehian-Dehkordi H, Esmailizadeh A, Nosrati M, Paiva S, Caetano A, Stepanek O, Olsaker I, Weimann C, Erhardt G, Curik I, Kantanen J, Mwacharo J, Hanotte O, Bruford M, Ciani E, Periasamy K, Amills M, Lenstra J, Han J, Zhang H, Li L, and Li M. Paternal Origins and Migratory Episodes of Domestic Sheep. Curr Biol 2020; 30:4085–4095.e6. DOI: 10.1016/j.cub.2020.07.077. Available from: https://www.ncbi.nlm.nih.gov/entrez/query.fcgi?db=PubMed&cmd=Retrieve&dopt=Citation&list_uids=32822607

15. Schwarz C, Debruyne R, Kuch M, McNally E, Schwarcz H, Aubrey A, Bada J, and Poinar H. New insights from old bones: DNA preservation and degradation in permafrost preserved mammoth remains. Nucleic Acids Res 2009; 37:3215–29. DOI: 10.1093/nar/gkp159. Available from: https://www.ncbi.nlm.nih.gov/entrez/query.fcgi?db=PubMed&cmd=Retrieve&dopt=Citation&list_uids=19321502

16. Dabney J, Knapp M, Glocke I, Gansauge MT, Weihmann A, Nickel B, Valdiosera C, García N, Pääbo S, Arsuaga JL, et al. Complete mitochondrial genome sequence of a Middle Pleistocene cave bear reconstructed from ultrashort DNA fragments. Proceedings of the National Academy of Sciences 2013 :201314445. DOI: 10.1073/pnas.1314445110

17. Glocke I and Meyer M. Extending the spectrum of DNA sequences retrieved from ancient bones and teeth. Genome Research 2017. DOI: 10.1101/gr.219675.116

18. Meyer M and Kircher M. Illumina sequencing library preparation for highly multiplexed target capture and sequencing. Cold Spring Harb Protoc 2010; 2010:pdb.prot5448. DOI: 10.1101/pdb.prot5448. Available from: https://www.ncbi.nlm.nih.gov/entrez/query.fcgi?db=PubMed&cmd=Retrieve&dopt=Citation&list_uids=20516186

19. Kircher M, Sawyer S, and Meyer M. Double indexing overcomes inaccuracies in multiplex sequencing on the Illumina platform. Nucleic Acids Res 2012; 40:e3. DOI: 10.1093/nar/gkr771. Available from: https://www.ncbi.nlm.nih.gov/entrez/query.fcgi?db=PubMed&cmd=Retrieve&dopt=Citation&list_uids=22021376

20. Hider J, Duggan AT, Klunk J, Eaton K, Long GS, Karpinski E, Giuffra V, Ventura L, Fornaciari A, Fornaciari G, Golding GB, Prowse TL, and Poinar HN. Examining pathogen DNA recovery across the remains of a 14th century Italian friar (Blessed Sante) infected with *Brucella melitensis*. International Journal of Paleopathology 2022; 39:20–34. DOI: https://doi.org/10.1016/j.ijpp.2022.08.002. Available from: https://www.sciencedirect.com/science/article/pii/S1879981722000420

21. Long G, Klunk J, Duggan A, Tapson M, Giuffra V, Gazze L, Fornaciari A, Duchene S, Fornaciari G, Clermont O, Denamur E, Golding G, and Poinar H. A 16(th) century *Escherichia coli* draft genome associated with an opportunistic bile infection. Commun Biol 2022; 5:599. DOI: 10.1038/s42003-022-03527-1. Available from: https://www.ncbi.nlm.nih.gov/entrez/query.fcgi?db=PubMed&cmd=Retrieve&dopt=Citation&list_uids=35710940

22. Hyatt D, Chen G, Locascio P, Land M, Larimer F, and Hauser L. Prodigal: prokaryotic gene recognition and translation initiation site identification. BMC Bioinformatics 2010; 11:119. DOI: 10.1186/1471-2105-11-119. Available from: https://www.ncbi.nlm.nih.gov/entrez/query.fcgi?db=PubMed&cmd=Retrieve&dopt=Citation&list_uids=20211023

23. Lagesen K, Hallin P, Rodland E, Staerfeldt H, Rognes T, and Ussery D. RNAmmer: consistent and rapid annotation of ribosomal RNA genes. Nucleic Acids Res 2007; 35:3100–8. DOI: 10.1093/nar/gkm160. Available from: https://www.ncbi.nlm.nih.gov/entrez/query.fcgi?db=PubMed&cmd=Retrieve&dopt=Citation&list_uids=17452365

24. Laslett D and Canback B. ARAGORN, a program to detect tRNA genes and tmRNA genes in nucleotide sequences. Nucleic Acids Res 2004; 32:11–6. DOI: 10.1093/nar/gkh152. Available from: https://www.ncbi.nlm.nih.gov/entrez/query.fcgi?db=PubMed&cmd=Retrieve&dopt=Citation&list_uids=14704338

25. Petersen T, Brunak S, vonHeijne G, and Nielsen H. SignalP 4.0: discriminating signal peptides from transmembrane regions. Nat Methods 2011; 8:785–6. DOI: 10.1038/nmeth.1701. Available from: https://www.ncbi.nlm.nih.gov/entrez/query.fcgi?db=PubMed&cmd=Retrieve&dopt=Citation&list_uids=21959131

26. Kolbe D and Eddy S. Fast filtering for RNA homology search. Bioinformatics 2011; 27:3102–9. DOI: 10.1093/bioinformatics/btr545. Available from: https://www.ncbi.nlm.nih.gov/entrez/query.fcgi?db=PubMed&cmd=Retrieve&dopt=Citation&list_uids=21965818

27. Page AJ, Cummins CA, Hunt M, Wong VK, Reuter S, Holden MT, Fookes M, Falush D, Keane JA, and Parkhill J. Roary: rapid large-scale prokaryote pan genome analysis. Bioinformatics 2015; 31:3691–3. DOI: 10.1007/978-3-642-37195-0_13

28. Suarez-Esquivel M, Chaves-Olarte E, Moreno E, and Guzman-Verri C. *Brucella* Genomics: Macro and Micro Evolution. Int J Mol Sci 2020; 21. DOI: 10.3390/ijms21207749. Available from: https://www.ncbi.nlm.nih.gov/entrez/query.fcgi?db=PubMed&cmd=Retrieve&dopt=Citation&list_uids=33092044

29. Moreno E. Genome evolution within the alpha Proteobacteria: why do some bacteria not possess plasmids and others exhibit more than one different chromosome? FEMS Microbiol Rev 1998; 22:255–75. DOI: 10.1111/j.1574-6976.1998.tb00370.x. Available from: https://www.ncbi.nlm.nih.gov/entrez/query.fcgi?db=PubMed&cmd=Retrieve&dopt=Citation&list_uids=9862123

30. Moreno E, Blasco J, Letesson J, Gorvel J, and Moriyon I. Pathogenicity and Its Implications in Taxonomy: The *Brucella* and *Ochrobactrum* Case. Pathogens 2022; 11. DOI: 10.3390/pathogens11030377. Available from: https://www.ncbi.nlm.nih.gov/entrez/query.fcgi?db=PubMed&cmd=Retrieve&dopt=Citation&list_uids=35335701

4 Tables

4.1 Tables

Table S1: **Marginal Likelihood Estimates for the Bayesian Phylogenies.** Marginal likelihood estimates for the Western Mediterranean and Global *B. melitensis* phylogenies. Cells shaded in green indicate the best model for either phylogeny.

	**Global**		**Western Mediterranean**
**Model**	**Strict Clock**	**Relaxed Clock**	**Strict Clock**
**No Dates**	−4,732,437.331	−4,732,205.307	−4,588,877.677
**Dated**	−4,732,381.306	−4,732,214.221	−4,588,817.929

Table S2: **Sequence Typing Results for the ancient *B. melitensis* assembly**

**ST**	*gap*	*aroA*	*glk*	*dnaK*	*gyrB*	*trpE*	*cobQ*	*int hyp*	*omp25*
NF	3	–	3	2	1	5	3	2	10

Table S3: **Comparison of Gene Content Analysis Methods.** The genetic content of the ancient *B. melitensis* genome was analysed using both an assembly approach and a mapping approach [21]. The mapping analysis was tested using either a -2σ or -3σ threshold from the mean read depth of the pan-genome.

	denovo **Assembly**	**Pan-Genome Mapping**	
**Metric**		-2σ	-3σ
**Genes Identified**	2996	2864	2995
**Core Genes**	2826	2656	2744
**Proportion of Core Genome** (%)	97.5	91.5	94.5
**Accessory Genes**	170	208	221
**Unique Genes**	39	0	0
